# GDP-mannose pyrophosphorylase is an efficient target in *Xanthomonas citri* for citrus canker control

**DOI:** 10.1128/spectrum.03673-23

**Published:** 2024-05-09

**Authors:** André Vessoni Alexandrino, Mariana Pegrucci Barcelos, Leonardo Bruno Federico, Tamiris Garcia da Silva, Lúcia Bonci Cavalca, Carlos Henrique Alves de Moraes, Henrique Ferreira, Carlton Anthony Taft, Franklin Behlau, Carlos Henrique Tomich de Paula Silva, Maria Teresa Marques Novo-Mansur

**Affiliations:** 1 Laboratório de Bioquímica e Biologia Molecular Aplicada (LBBMA), Departamento de Genética e Evolução, Universidade Federal de São Carlos, São Carlos, São Paulo, Brazil; 2 Programa de Pós-Graduação em Biotecnologia (PPGBiotec), Universidade Federal de São Carlos, São Carlos, São Paulo, Brazil; 3 Faculdade de Ciências Farmacêuticas de Ribeirão Preto, Universidade de São Paulo, Ribeirão Preto, São Paulo, Brazil; 4 Departamento de Pesquisa e Desenvolvimento, Fundo de Defesa da Citricultura, Fundecitrus, Araraquara, São Paulo, Brazil; 5 Departamento de Bioquímica e Microbiologia, Instituto de Biociências, UNESP, Universidade Estadual Paulista, Rio Claro, São Paulo, Brazil; 6 Centro Brasileiro de Pesquisas Físicas, Rio de Janeiro, Brazil; 7 Programa de Pós-Graduação em Genética Evolutiva e Biologia Molecular (PPGGEv), Universidade Federal de São Carlos, São Carlos, São Paulo, Brazil; USDA - San Joaquin Valley Agricultural Sciences Center, Parlier, California, USA

**Keywords:** *Xanthomonas *, citrus canker, GDP-mannose pyrophosphorylase, phosphomannose isomerase, inhibitors, citrus protection, innovation

## Abstract

*Xanthomonas citri* subsp. *citri* (Xcc) is a bacterium that causes citrus canker, an economically important disease that results in premature fruit drop and reduced yield of fresh fruit. In this study, we demonstrated the involvement of XanB, an enzyme with phosphomannose isomerase (PMI) and guanosine diphosphate-mannose pyrophosphorylase (GMP) activities, in Xcc pathogenicity. Additionally, we found that XanB inhibitors protect the host against Xcc infection. Besides being deficient in motility, biofilm production, and ultraviolet resistance, the *xanB* deletion mutant was unable to cause disease, whereas *xanB* complementation restored wild-type phenotypes. XanB homology modeling allowed *in silico* virtual screening of inhibitors from databases, three of them being suitable in terms of absorption, distribution, metabolism, excretion, and toxicity (ADME/Tox) properties, which inhibited GMP (but not PMI) activity of the Xcc recombinant XanB protein in more than 50%. Inhibitors reduced citrus canker severity up to 95%, similarly to copper-based treatment. *xanB* is essential for Xcc pathogenicity, and XanB inhibitors can be used for the citrus canker control.

**IMPORTANCE:**

Xcc causes citrus canker, a threat to citrus production, which has been managed with copper, being required a more sustainable alternative for the disease control. XanB was previously found on the surface of Xcc, interacting with the host and displaying PMI and GMP activities. We demonstrated by *xanB* deletion and complementation that GMP activity plays a critical role in Xcc pathogenicity, particularly in biofilm formation. XanB homology modeling was performed, and *in silico* virtual screening led to carbohydrate-derived compounds able to inhibit XanB activity and reduce disease symptoms by 95%. XanB emerges as a promising target for drug design for control of citrus canker and other economically important diseases caused by *Xanthomonas* sp.

## INTRODUCTION

Citrus canker is a disease caused by the bacterium *Xanthomonas citri* subsp. *citri* (Xcc), which manifests as circular, raised, cortical, and brown lesions on leaves, branches, and fruits (1). In more severe cases, it can lead to premature fruit drop, directly impacting citrus production (2). In citrus-growing regions where the disease is endemic, a range of control measures is employed to minimize crop loss, including the use of resistant or less susceptible cultivars, windbreaks around the orchard perimeter, and frequent application of copper-based bactericides during spring and summer months (3, 4). While copper-based treatments have shown effectiveness in managing citrus canker, their long-term use may have negative environment implications and pose potential risks to consumers, highlighting the necessity for the development of novel disease management strategies.

Previous studies carried out at Laboratory of Biochemistry and Applied Molecular Biology (from the Portuguese "Laboratório de Bioquímica e Biologia Molecular Aplicada, LBBMA") at the Federal University of São Carlos by Carnielli and colleagues (5) demonstrated a 4.8-fold increase in the abundance of the XanB enzyme on the surface of Xcc cells grown *in vivo* compared to those grown *in vitro*. This enzyme is associated with the production of xanthan gum, an exopolysaccharide known to play a crucial role in bacterial defense by protecting cells against dehydration in harsh environments (6), as well as in pathogen-host interactions (7).

XanB is a metalloenzyme with phosphomannose isomerase (PMI) or mannose-6-phosphate isomerase activity, catalyzing the interconversion of fructose-6-phosphate and mannose-6-phosphate. Additionally, the enzyme possesses GDP-mannose pyrophosphorylase (GMP) activity, which catalyzes the synthesis of guanosine mannose diphosphate (GDP-mannose) (8). The putative PMI-GMP enzyme encoded by the ORF XAC3580 in Xcc [National Center for Biotechnology Information (NCBI)] is bifunctional, known as type II (9), consists of 467 amino acid residues, and belongs to the family of mannose-1-phosphate guanylyltransferase and mannose-6-phosphate isomerase (10). Notably, this enzyme is conserved in various other microorganisms and serves as a potential therapeutic target in animal pathogens such as *Candida albicans* (11), *Mycobacterium smegmatis* (12), and *Leishmania mexicana* (13).

Computational chemistry tools have been employed in designing novel drugs that interact with specific targets, aiming to prevent the progression of diseases (14, 15). Virtual screening is a commonly used computational technique for drug discovery, which identifies compounds capable of binding to a particular protein or enzyme (16). This approach involves two main methods: ligand-based and structure-based methods (17). The ligand-based method analyzes electronic, structural, physicochemical, and molecular similarities between different ligands and suggests a binding mechanism to a specific receptor (18). Ligand similarity methods are based on the assumption that compounds similar to a known active ligand for a given target are more likely to be active than those without similar characteristics (19). Structure-based methods include computational approaches to analyze the structure of the target. One of the most used techniques within this method is molecular docking, which employs a scoring function to indicate ligands with greater affinity for the active binding site (20, 21).

Since the 1980s, computational techniques have been utilized for drug discovery, particularly for human consumption (22). However, there is significant potential to develop novel and safer products for disease and pest control in agriculture. Therefore, this study aims to demonstrate the essential role of GDP-mannose pyrophosphorylase activity of XanB in the pathogenicity of Xcc and to screen and evaluate inhibitors of this activity as sustainable alternative to copper for protecting against citrus canker.

## RESULTS

### *xanB* is an essential gene for Xcc pathogenicity in *Citrus aurantifolia*

The colonies of the deletion mutant and complemented strains (XccΔxanB and XccΔCxanB, respectively) selected on sucrose were assessed for gene deletion and complementation by PCR. The amplicons were of the expected sizes, being 3.5 kb for the wild type (Fig. S1, lane 1) and complemented strains (Fig. S1, lane 7), and 2 kb for the mutant strain (Fig. S1, lane 2). As expected, no PCR amplification was obtained using the pNPTS138_*xanB* deletion vector as a template (Fig. S1, lane 4). To confirm that the amplified PCR product from these strains corresponded to the chromosomal regions of interest, digestion with *EcoR*I was performed, which resulted in two 1-kb bands for XccΔxanB (Fig. S1, lane 3) and a 1.5-kb band for the coding region of the *xanB* gene, and another 1-kb band referring to the two flanking regions of 1 kb each for XccΔCxanB (Fig. S1, lane 8).

**Fig 1:**
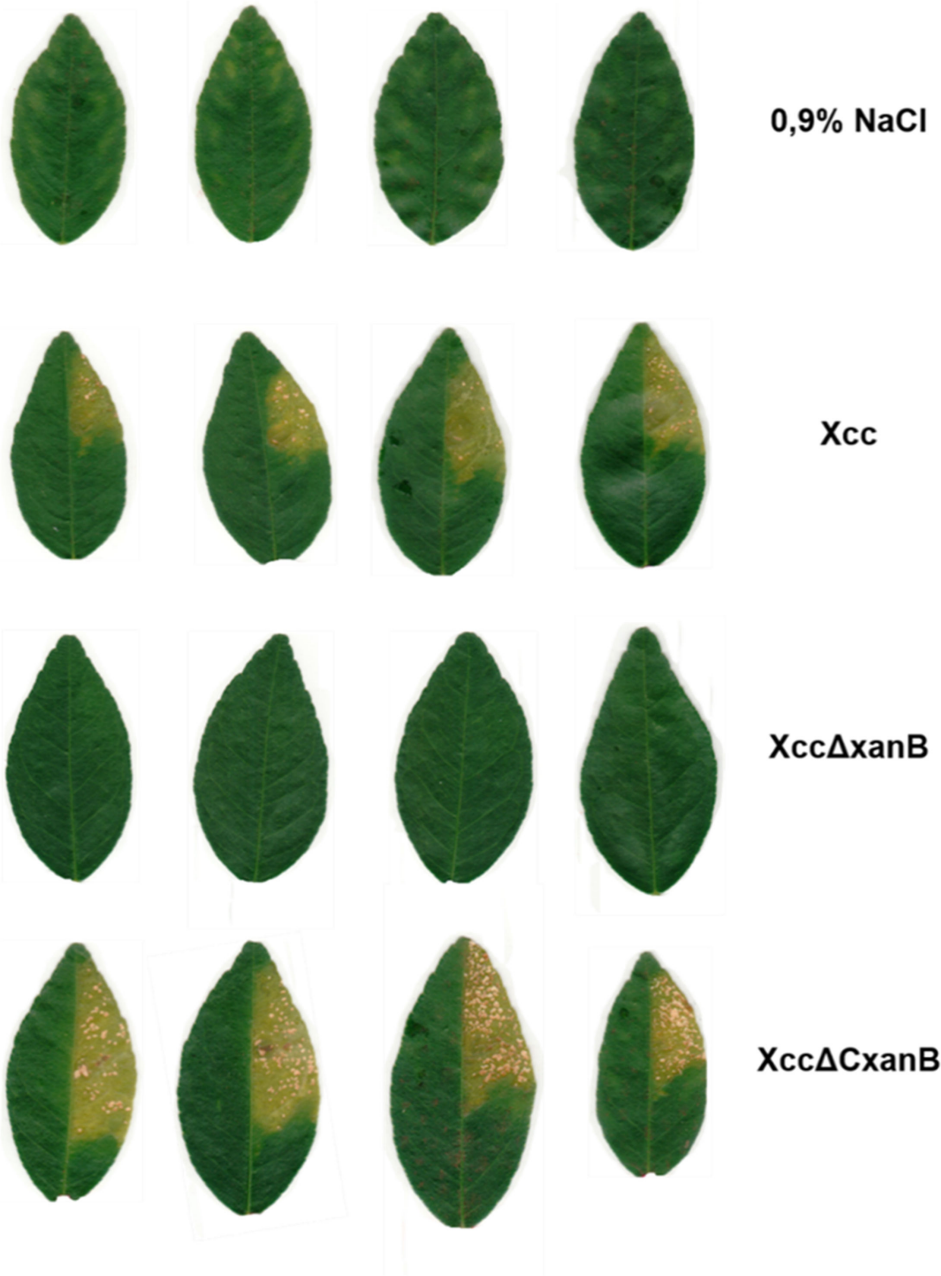
*In vivo* pathogenicity assay by infiltration of Xcc, XccΔxanB, and XccΔC*xanB* into *Citrus aurantifolia* leaves. Plants of *C. aurantifolia* were utilized for comparative evaluation of pathogenicity and virulence of Xcc, XccΔxanB, and XccΔCxanB. The positive control image (Xcc) is identical to that used in our previous work for the *in vivo* assays for *xylA* gene mutants (23), since both assays were carried out together. All plants had four leaves of independent branches infiltrated with the three strains and the negative control using saline (0.9% NaCl). Leaves were documented 20 days post-inoculation.

**Fig 2:**
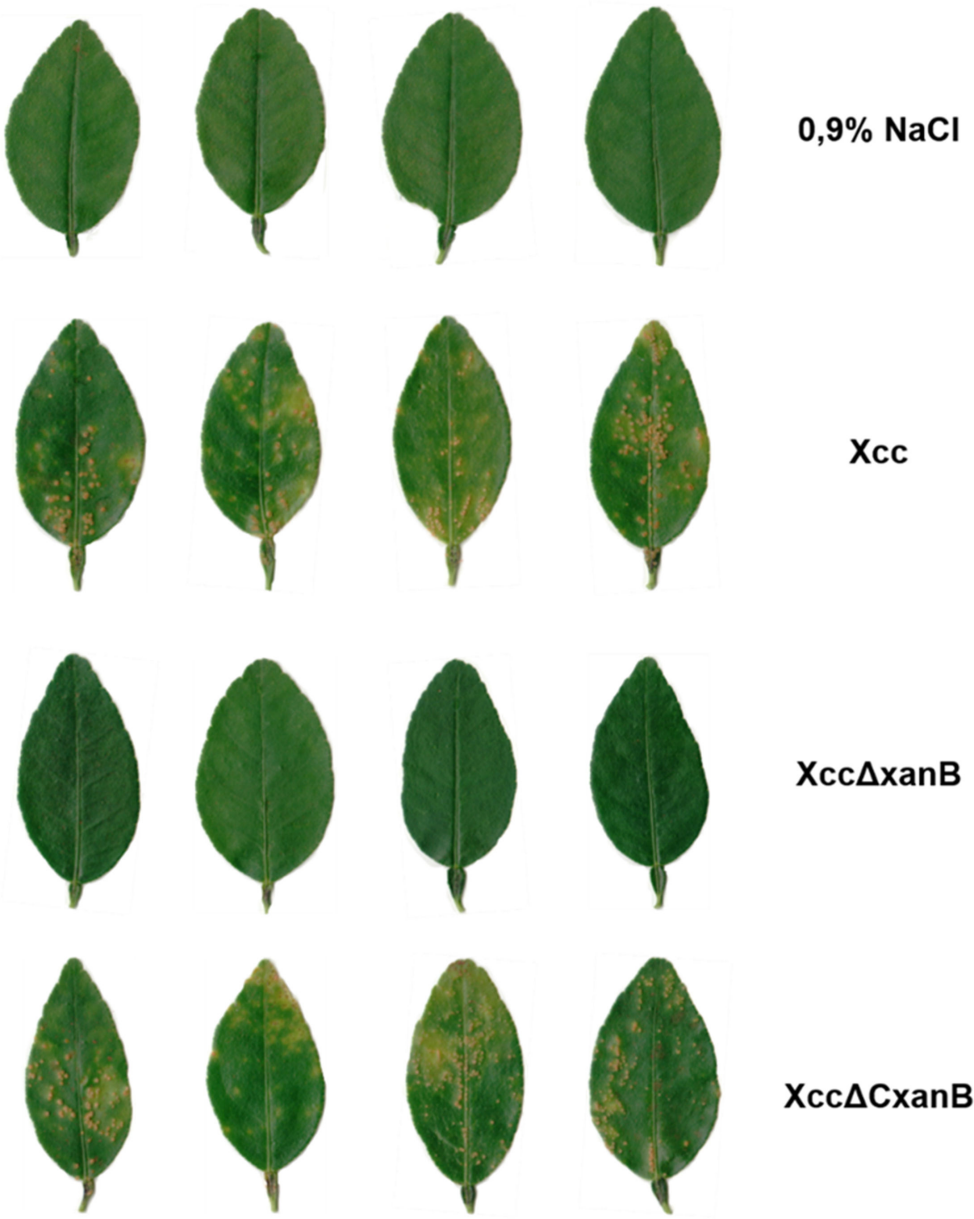
*In vivo* pathogenicity assay by spraying of Xcc, XccΔxanB, and XccΔC*xanB* in *Citrus aurantifolia* leaves. Potted *Citrus aurantifolia* plants kept in a greenhouse were used for comparative evaluation of the pathogenicity and virulence of Xcc, XccΔxanB, and XccΔCxanB. Ten milliliters of each bacterial culture and the same volume of saline solution (negative control) were sprayed on four plants. Leaves were scanned 28 days post-inoculation.

The mutant, complemented, and wild-type strains were evaluated for pathogenicity and virulence in *Citrus aurantifolia* through syringe infiltration and spraying. Twenty days post-infiltration, the leaves were detached and digitally recorded to compare the infection (Fig. 1). In the spray test, the bacterial suspension and saline solution were each sprayed, and leaves were digitally recorded 28 days post-spraying (Fig. 2).

The wild-type Xcc strain induced the typical citrus canker symptoms, as expected, whereas the deletion of *xanB* reduced pathogenicity for XccΔxanB in both infiltration and spray inoculation. Furthermore, the complemented strain XccΔCxanB showed a phenotype reversion. Both Xcc and XccΔCxanB strains caused similar degrees of water soaking and disease severity (Fig. 1 and 2).

### *xanB* gene is involved in motility, biofilm formation, and UV resistance of Xcc

**Fig 3:**
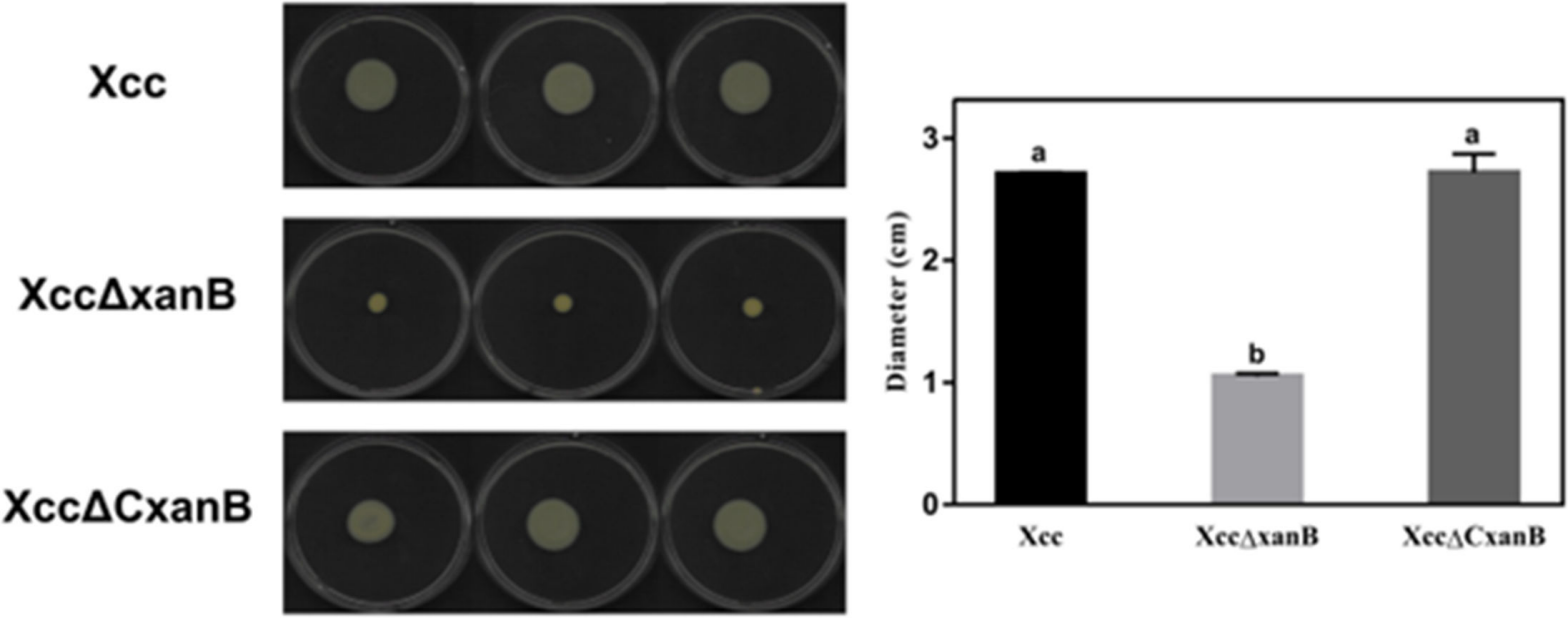
Motility of xcc, XccΔxanB, and XccΔC*xanB*. Cultures were pipetted in the center of petri dishes with 0.7% Luria-Bertani agar. Plates were incubated at 30°C for 48 h without shaking and were digitally recorded (on the left). The diameter of the colonies obtained was measured using ImageJ software. The calculated averages are presented graphically (on the right). Error bars indicate the absolute standard deviation of each of the triplicates. Columns followed by the same letter do not show significant difference using Tukey’s test (*P =* 0.05).

The motility capacity was evaluated by measuring the diameter of the colonies, which were 2.70 ± 0.02, 1.15 ± 0.02, and 2.72 ± 0.09 cm for Xcc, XccΔxanB, and XccΔCxanB, respectively (Fig. 3). XccΔxanB showed a reduction in motility of approximately 60% compared to wild-type Xcc and the complemented strain XccΔCxanB, indicating that the *xanB* gene plays a role in this characteristic related to bacterial pathogenicity.

**Fig 4:**
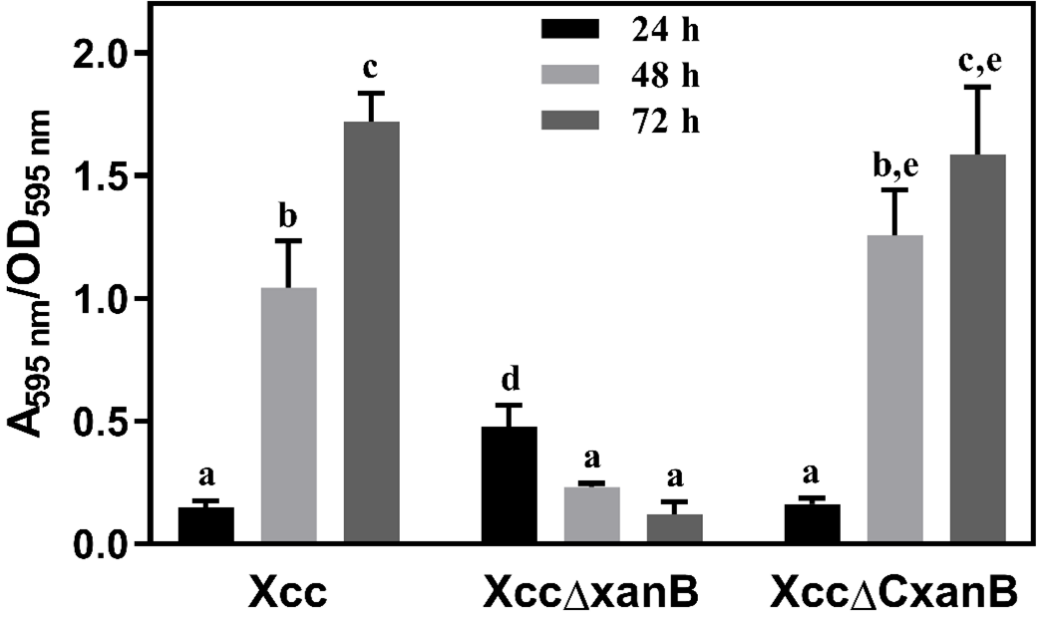
Biofilm formation by Xcc, XccΔxanB, and XccΔC*xanB*. Cultures were evaluated for biofilm formation. The results are presented as the average of the ratio between the crystal violet absorbance at 595 nm and the optical density of each bacterial culture immediately before the measurement of biofilm formation (*A*_595 nm_/OD_595 nm_). Measurements were taken at 24, 48, and 72 h after incubation. Error bars indicate the absolute standard deviation of each of the sextuplicates. Columns followed by the same letter do not show significant difference using Tukey’s test (*P* = 0.05).

The three strains were evaluated for biofilm formation over 24, 48, and 72 h. After 24 h, the biofilm produced by XccΔxanB was higher than that of Xcc and XccΔCxanB. However, after 48 and 72 h, this pattern was significantly reversed, with biofilm formation in XccΔxanB being 4.5 and 14.5 times lower, respectively, than in Xcc (Fig. 4). The biofilm produced by XccΔCxanB was similar to that of Xcc, confirming the effect of *xanB* depletion on biofilm formation in XccΔxanB.

**Fig 5:**
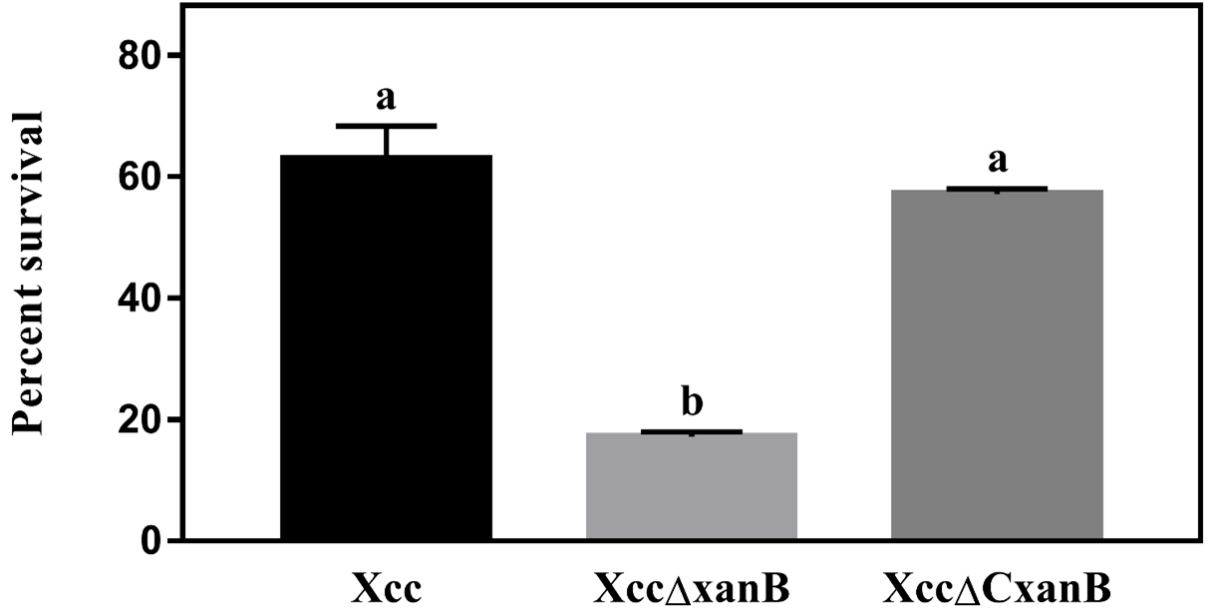
Evaluation of resistance to ultraviolet (UV) radiation by Xcc, XccΔxanB, and XccΔCxanB. Cultures were evaluated for survival after exposure to UV radiation. The results are presented in percentage of survival relatively to the controls of each bacterial strain not exposed to UV radiation. Error bars indicate the absolute standard deviation of each of the triplicates. Columns followed by the same letter do not show significant difference using Tukey’s test (*P =* 0.05).

The *xanB* gene also plays a role in Xcc resistance to ultraviolet (UV) radiation. XccΔxanB was found to survive for a period three times shorter than Xcc. The wild-type level of UV resistance was restored in XccΔCxanB through *xanB* complementation (Fig. 5).

### XanB is a bifunctional enzyme

*Escherichia coli* BL21 (DE3) transformed with the pET41a_*xanB* vector and induced with isopropyl-ß-D-thiogalactopyranoside (IPTG) is expected to produce an 85-kDa recombinant protein fused to glutathione S-transferase (GST), with 51 and 26 kDa corresponding to the *xanB* and GST coding regions, respectively, and an additional 8-kDa fragment encoded by the pET41a vector. SDS-PAGE analysis showed that the recombinant protein was predominantly present in the soluble fraction (Fig. S2).

The isomerase activity of the recombinant XanB was confirmed using Seliwanoff’s reagent (Fig. S3) and a coupling method with PGI and G6PD (Fig. S4). The second predicted activity of the recombinant XanB was also confirmed by the pyrophosphatase coupling method (Fig. S5).

### Xcc XanB tridimensional modeling and *in silico* searching for XanB inhibitors

#### Construction and evaluation of Xcc XanB and human PMI homology models

In the absence of a deposited Xcc XanB crystallographic structure in the Protein Data Bank, a homology model was employed to discover novel potential inhibitors using a structure-based drug design. To construct the homology model for Xcc XanB, the National Institutes of Health BLASTp server (24) was utilized, which identified the following homologous structures with high-resolution crystallographic data (ranging from 1.9 to 2.35 Å): PDB IDs 2CU2, 2 × 5S and 2QH5, sharing 38%, 40%, and 40% sequence identity, respectively, with Xcc XanB. Additionally, the PDB ID 1H5R, with 29% sequence identity and containing a ligand (the substrate) located within the enzyme’s active site, was also considered/selected.

The selection of these crystallographic structures as templates in the homology modeling process included a PMI structure in complex with a ligand, specifically, the B chain of the PDB ID 1H5R protein structure. Given that all the selected structures share above 25% sequence identities with Xcc XanB, they are likely to exhibit potential structural and functional similarities with the modeled structure.

Based on the sequences of the Xcc XanB homologs used as templates, we performed a multiple alignment analysis using the referred templates and Xcc XanB. Sequence alignments were conducted using the Clustal Omega web server (25), and the four crystallographic structures mentioned above were superimposed using the Discovery Studio software to verify the quality of the alignment.

When comparing both alignments, one obtained using only Clustal Omega and the other refined, some differences were observed. Certain gaps were suggested based on the templates’ structural superpositions, with the most evident one occuring in the PDB ID 2QH5 structure, where a gap was added between the Gly11 and Tyr12 amino acid residues (Fig. S6).

Finally, the MODELLER-generated final model was validated using WHAT IF and PROCHECK, including the Ramachandran Plot, Verify-3D, ERRAT, and PROVE methodologies (described in the supplemental material). The results confirmed the suitability of the models in structure-based virtual screening (SBVS) simulations.

A human PMI model was also constructed and validated using the same protocol as the Xcc XanB model to facilitate the evaluation and comparison of the two enzymes’ active sites, which are distinct. This approach enabled us to obtain specific inhibitors for Xcc XanB.

#### Model and methodology validation

**Fig 6:**
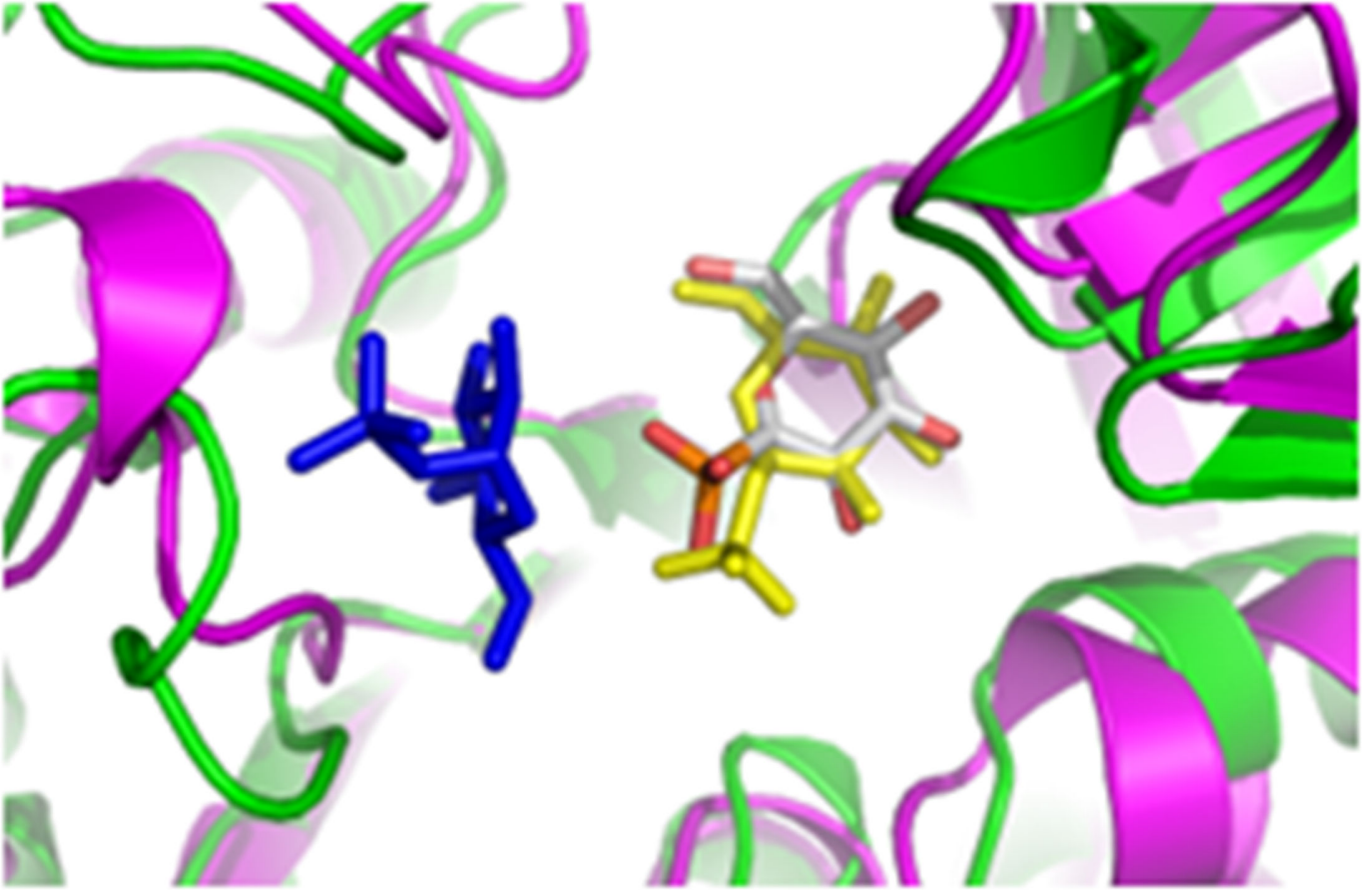
Superposition between the Xcc XanB model (ribbon representation in pink) and the PDB ID 1H5R structure (ribbon representation in green). The figure shows the docking poses of the glucose-1-phosphate ligand in comparison to the crystallographic pose (stick representation, colored by atoms), inside the XanB GMP active site, when using the GOLD (stick representation, in yellow) and GLIDE (stick representation, in blue) software.

We performed a redocking study (26) to select the appropriate docking program for our simulations (GOLD and GLIDE). The program that demonstrated the highest pose conformity and an root-mean-square deviation (RMSD) below 2.5 Å, which is considered a partial success by the aforementioned authors (27, 28), was GOLD (Fig. 6). Therefore, GOLD was selected for our future docking simulations in comparison with the crystallographic ligand (glucose-1-phosphate).

#### Models’ validation

After selecting and refining the final Xcc XanB model, we evaluated its efficiency for virtual screening studies using the receiver operator characteristic curve (ROC) and area under the curve (AUC) (29, 30). A database was constructed using 30 ligands with reported biological activity values (IC_50_) from the BindingDB web server (31) (supplemental material).The AUC ROC value obtained for this study with our model was 0.760, which was close to the ideal value of 1.

For human PMI, the studies also showed favorable results, with an obtained AUC value of 0.792, which is considered significant when close to the ideal value of 1.

Therefore, based on these validation results, we can confirm that the models built for both Xcc and human PMI are capable of distinguishing between more and less potent XanB inhibitors selected from the BindingDB structures repository. Furthermore, the Xcc model is suitable for subsequent virtual screening studies. Additional information about the model validation can be found in the supplemental material.

#### Virtual screening procedures

For virtual screening purposes, we divided the compound databases into two groups. The first group consisted of ZINC, Drugs FDA, Chembridge and Maybridge, while the second group included the Princeton and IBS databases. In the virtual screening simulations involving compounds from the first group, we selected the 2,000 top-ranked compounds from each database. These compounds were obtained by virtual screening based on shape similarity and were scored according to their Tanimoto similarity indexes (ROCS_TanimotoCombo). Then, we retrieved the 1,000 top-ranked results for each base based on the Tanimoto index (EON_ET_Combo), resulting in a total of 7,000 surviving compounds after the first stage of our protocol.

The compounds obtained in the previous step underwent docking simulations, and the 500 top-ranked ones were selected based on the GOLD Fitness function for each database. Subsequently, these compounds had their ADME/Tox properties predicted, resulting in 117 compounds in this first group of compound databases. The next stage involved visual inspection of the binding modes predicted for these compounds in relation to the Xcc XanB model, from which only 40 molecules were selected as potential and promising candidates for Xcc XanB inhibition.

**Fig 7:**
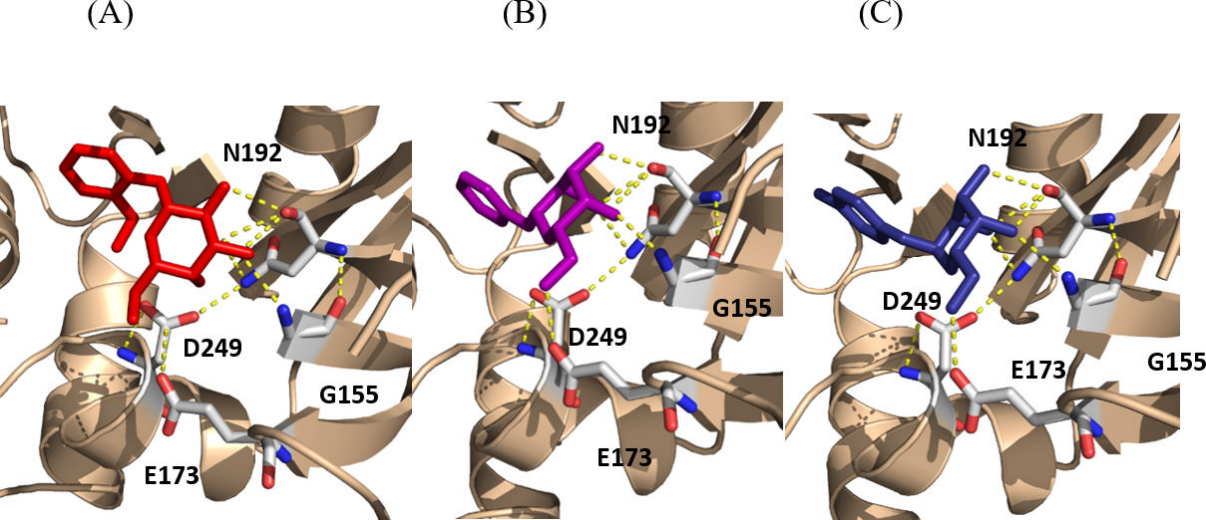
Stick representation of docking poses for the selected inhibitors i1 (**A**), i2 (**B**), and i3 (**C**) inside the Xcc XanB (in ribbon representation) GMP active site, with hydrogen bonds represented in yellow dashed lines. Main residues of the XanB active site are depicted here with labels.

Finally, 33 compounds were commercially acquired, and three compounds emerged as the most promising, referred to as i1, i2, and i3. According to our docking simulations, these three selected inhibitors potentially interact with the same amino acid residues (Gly155, Glu173, Asn192, and Asp249). Two of them (i1 and i2) establish all these interactions, with Gly155 acting as a hydrogen bond donor, while the other amino acids (Glu173, Asn192, and Asp249) act as hydrogen bond acceptors (Fig. 7).

The other compound, i3, demonstrates potential interactions with Gly155, serving as a hydrogen bond donor, though its interactions with other amino acid residues, such as Glu173 and Asn192, are distinct, excluding any interaction with the Asp249 residue (Fig. 7). In the context of virtual screening simulations for compounds from the second group, we initially identified the top 5,000 compounds using similarity-based virtual screening focused on the shape of the ligand. These compounds were evaluated based on their Tanimoto similarity indexes, leading to the selection of the top 3,000 compounds by this metric. Following this, docking simulations were conducted on these compounds, and the 500 with the highest scores, as determined by the GOLD Fitness function, were chosen from each database. The ADME/Tox properties of these compounds were then predicted, narrowing the field to 13 promising candidates from this second group of compound databases. A final visual inspection of these molecules’ potential binding modes with the Xcc XanB model was performed, yet none of the compounds met the criteria to proceed beyond this stage of the protocol.

### Pyrophosphorylase activity of the recombinant XanB is inhibited by the selected compounds

**Fig 8:**
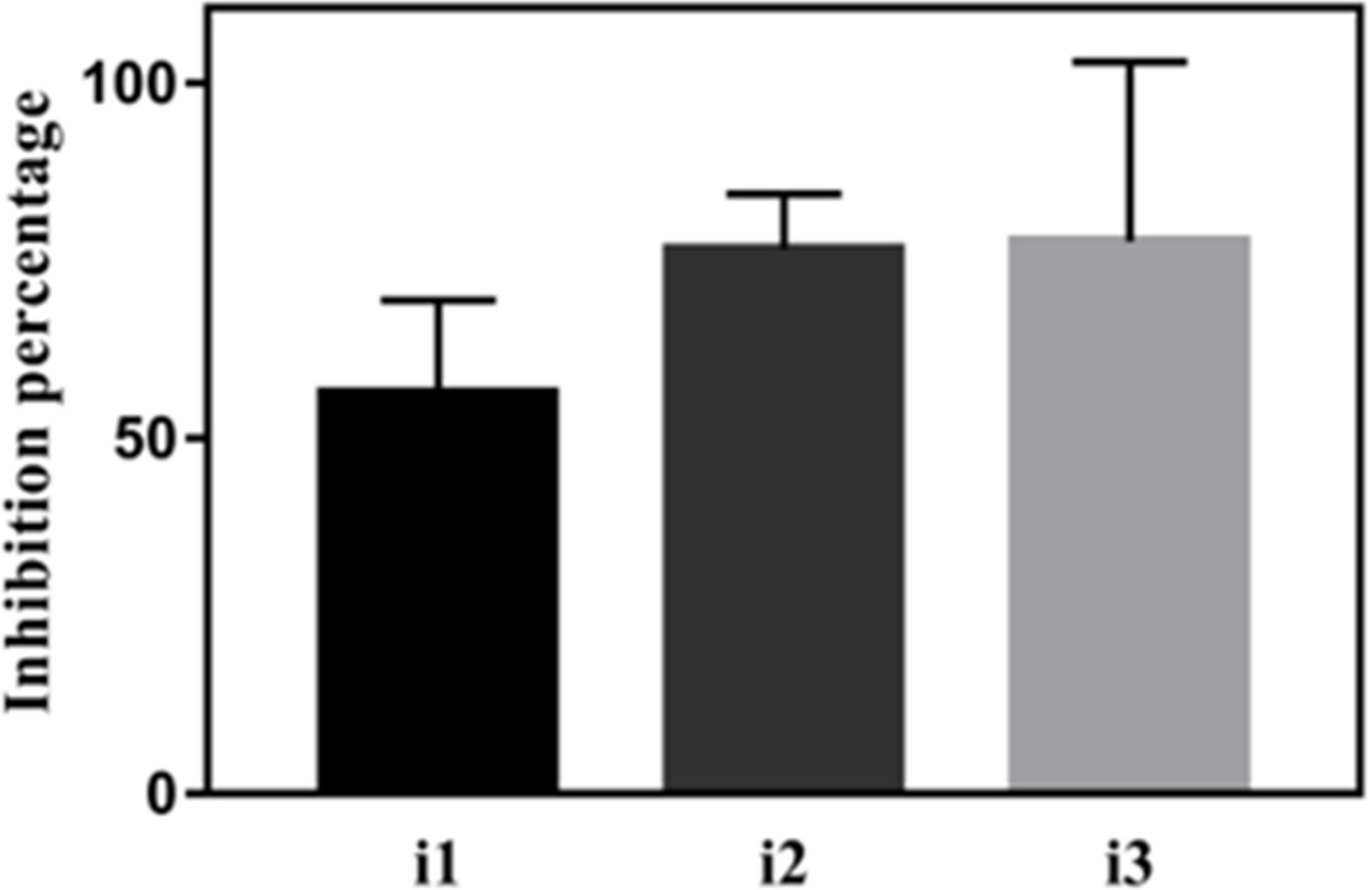
Pyrophosphorylase activity of the recombinant XanB in the presence of the potential XanB inhibitors. *In silico* selected inhibitors i1, i2, and i3 at 1 mM were tested for the ability to inhibit the pyrophosphorylase activity of the recombinant XanB. The inhibition percentage was calculated in relation to the reaction that replaces the volume of inhibitors by buffer 50-mM Tris-HCl, pH 8.0, and 100-mM NaCl.

The inhibition assay for the pyrophosphorylase activity of recombinant XanB confirmed the effectiveness of the three compounds at 1 mM, with inhibition percentages of 54.96% (SD = 12.66, *P* = 0.035), 74.11% (SD = 6.63, *P* = 0.0042) and 78.01% (SD = 21.32, *P* = 0.0038) for i1, i2, and i3, respectively (Fig. 8). There was no statistical difference in the inhibition percentages when comparing the three compounds.

Compounds i1 and i2 were also tested for their ability to inhibit the isomerase activity of recombinant XanB (Fig. S7). For i3, it was not possible to determine its inhibitory effect due to its strong absorbance at 340 nm (data not shown). The negative control, which did not include recombinant XanB, exhibited lower absorbance at 340 nm than the reaction containing the enzyme (enzyme test) (*P* < 0.0001), as previously observed (Fig. S4). However, there were no significant differences between the positive control (without inhibitor) and the reactions containing i1 (*P* = 0.4318) and i2 (*P* = 0.9984), indicating that both compounds do not inhibit the isomerase activity of recombinant XanB.

**Fig 9:**
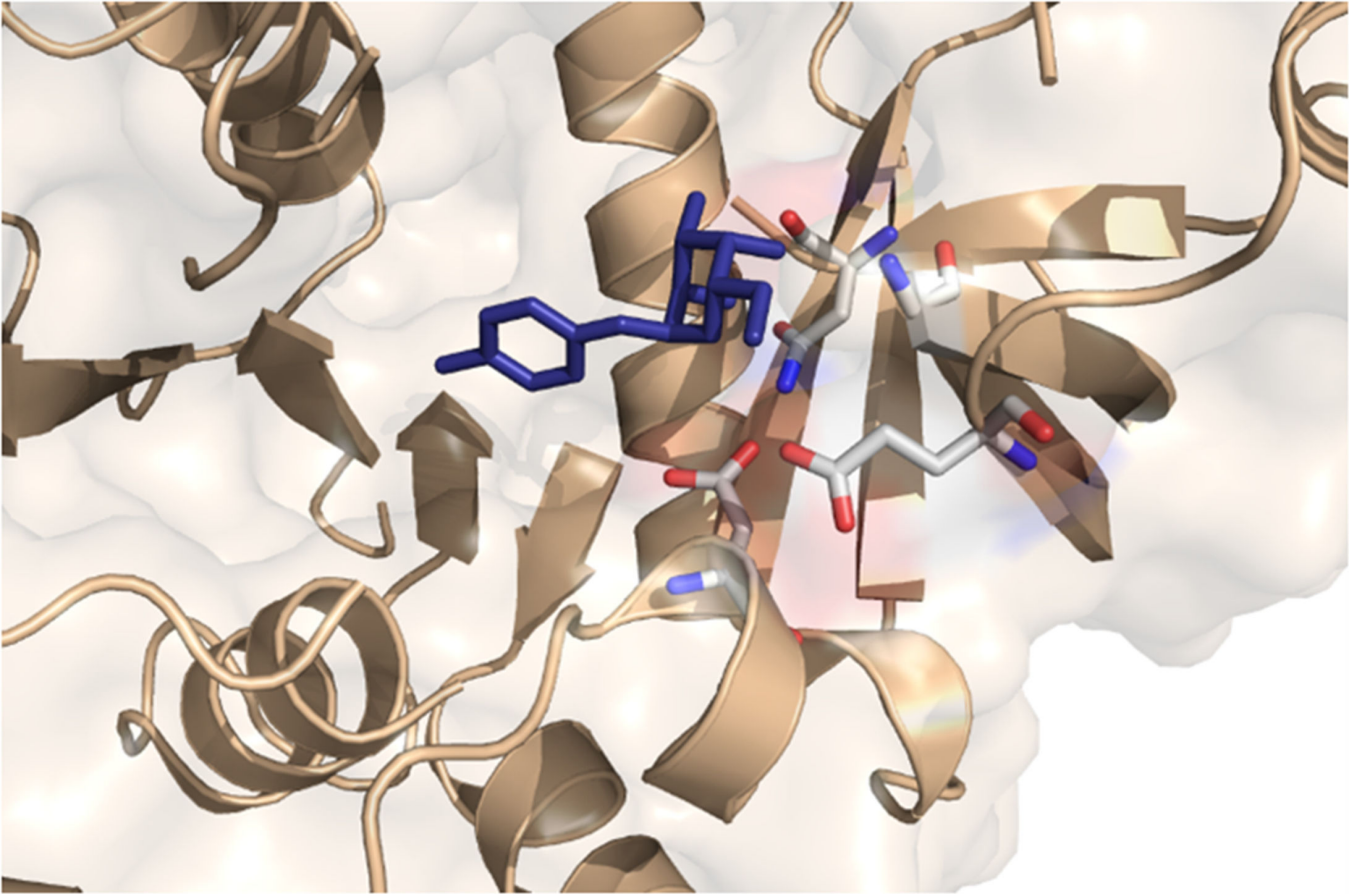
Surface, stick, and ribbon representations of i3 located inside the XanB active site. In this figure, the i3 inhibitor is visualized in stick representation (atoms in blue); selected XanB GMP active site residues are visualized in stick representation (colored by atoms); and PMI is visualized in surface as well as ribbon representations (in light brown).

The docking pose of the most potent inhibitor of the pyrophosphorylase activity of recombinant XanB investigated (i3) is represented inside the Xcc XanB structure (Fig. 9).

### XanB inhibitors protect *C. aurantifolia* and *C. sinensis* against citrus canker

**Fig 10:**
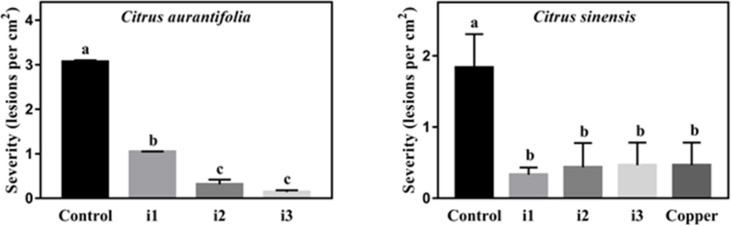
Potential of the inhibitors for protection of *C. aurantifolia* and *C. sinensis* against citrus canker. For each treatment, 10 leaves from three plants were sprayed on both surfaces with inhibitors i1, i2, i3, water (control), and copper oxychloride (the latter only for the assay with *C. sinensis*). The plants were spray-inoculated 24 h after the application of the inhibitors using Xcc suspension at 10^6^ CFU/mL and kept in a plastic humid chamber for 24 h in a humid room. Marked leaves from of each plant were evaluated 35 dpi, and the number of lesions on each leaf was divided by the leaf area. Error bars indicate the absolute standard deviation of each of the triplicates. Columns followed by the same letter do not show significant difference using Tukey’s test (*P* = 0.05).

The protective effect of XanB inhibitory compounds against Xcc was evaluated 35 days after spray inoculation of Xcc in *C. aurantifolia* and *C. sinensis* (Fig. 10). In both hosts, the three selected inhibitors reduced the severity of citrus canker, as indicated by the number of lesions per leaf area. In *C. aurantifolia*, inhibitor 3 reduced disease severity by 95.4% compared to the untreated control (*P* < 0.0001). For inhibitors 1 and 2, disease control reached 65.9% (*P* < 0.0001) and 90.4% (*P* < 0.0001), respectively.

In *C. sinensis*, the severity of citrus canker was reduced by inhibitors 1, 2, and 3 by 82.0% (*P* = 0.0018), 76.5% (*P* = 0.0031), and 74.9% (*P* = 0.0036), respectively. This level of control was similar to that provided by copper treatment on *C. sinensis*, which reduced the disease symptoms by 74.9% (*P* = 0.0036).

## DISCUSSION

In this study, we conducted functional characterization of the Xcc *xanB* gene, confirming its predicted enzymatic activities of PMI and GMP and demonstrating its essentiality for Xcc pathogenicity. Our research group previously identified XanB, encoded by the ORF XAC3580, as a potential contributor to pathogenicity through differential surface proteomics analysis of Xcc cells under *in vivo* and *in vitro* conditions (5). XanB was also identified in a proteomic analysis of *Xanthomonas campestris* pv*. campestris* interacting with *Brassica oleracea* (32). Additionally, we successfully selected XanB inhibitors capable of protecting *C. aurantifolia* and *C. sinensis* against citrus canker, further highlighting the potential of XanB as a target for disease control strategies (5, 33).

Deletion of a single gene resulting in the loss of pathogenicity is not so common in plant pathogens. Several mutant strains of Xcc have exhibited reduced virulence (32, 34–37). Deletion of *xanB* led to the inability of Xcc to induce symptoms in the host using both spray and infiltration methods (Fig. 1; Fig. 2). This suggests that the absence of symptoms is due to the inability not solely to survive in the epiphytic environment or to invade the host but also to colonize the apoplastic milieu. It is worth highlighting that the complemented strain XccΔCxanB regained its pathogenicity and virulence phenotypes, confirming that the loss of pathogenicity in XccΔxanB resulted from the deletion of *xanB*.

In our study, we observed increased severity of symptoms of the complemented strain relatively to the wild strain, which is an intriguing finding. This variation in disease severity could be attributed to several factors beyond mere biological variability. Environmental and experimental conditions, including slight differences in the age and health status of the plants as well as the microenvironment at the time of inoculation, may influence the expression of virulence factors and the host’s susceptibility to infection. Moreover, the influence of epigenetic factors on the virulence and genetic characteristics of bacterial plant pathogens has been documented (38). In constructing the complemented strain, we reintroduced the *xanB* gene into its original genomic *locus*, being that this copy has *Eco*RI flanking sites which were incorporated during construction of the plasmid used for complementation. These sites are absent in the wild-type strain, providing unique targets for Xcc *Eco*RI methylases in the complemented strain.

As demonstrated by Baránek and colleagues (38), the application of DNA demethylating chemicals has clear effects, with treated strains exhibiting reduced virulence. These findings suggest that methylation patterns can significantly affect the pathogenicity of Xcc strains, potentially explaining the observed increase in virulence of the complemented strain in our experiments relatively to the wild strain. This hypothesis suggests a nuanced understanding of pathogenicity, where modification of *xanB* vicinity can inadvertently affect virulence through epigenetic mechanisms.

In addition to the pathogenicity analyses previously described, growth tests were conducted to assess the impact of the *xanB* gene deletion on the growth capability of the XccΔxanB under nutrient-rich conditions. For this purpose, both the mutant and wild-type strains were cultured in a rich growth medium, such as Luria-Bertani (LB), and their growth was monitored (results not shown). The growth capacity of the *xanB* mutant strain was significantly affected, exhibiting a reduced growth rate compared to the wild-type strain. These findings indicate that the deletion of the *xanB* gene not only compromises Xcc pathogenicity but also adversely affects its growth in nutrient-rich environments. This observation suggests a potential role of the *xanB* gene in Xcc adaptation and survival under varying environmental conditions, in addition to its known role in pathogenicity.

The involvement of XanB in the synthesis of GDP-D-mannose, a precursor in the lipopolysaccharide (LPS) production pathways, emphasizes the already known importance of this exopolymer for the Xcc infection process. LPS constitutes the outer membrane of Gram-negative bacteria and acts as an elicitor molecule during plant-pathogen interactions. It triggers defense responses, including the generation of reactive oxygen species (39), the expression of defense-related genes (40), and the influx of cytosolic calcium (41), which induce programmed cell death (42).

The triggering of host defense responses is partially responsible for the appearance of symptoms during citrus canker pathogenesis. In *X. campestris* pv*. campestris*, interruption of the *xanB* gene with a transposon produced a strain with an altered profile of LPS, lacking the outer portion of the core sugar and O-antigen and an incomplete inner portion of the core sugar. As expected, the mutation caused the absence of mannose units in LPS and decreased the amounts of galacturonic acid and glucose. Consequently, the mutant strain elicited milder host defense responses, including oxidative burst (43), which can also be expected for XccΔxanB. Thus, it is possible to suggest that the absence of symptoms of XccΔxanB (Fig. 1; Fig. 2) is due to the host’s inability to recognize bacterial LPS, leading to a weak oxidative defense response. Although the ability to remain unrecognized by the host seems advantageous for XccΔxanB, this strain was not able to successfully infect the host, which can be explained by other factors.

Cell adhesion and biofilm formation are critical factors for the success of the Xcc infection process. XccΔxanB exhibited a 61.15% reduction in motility compared to the wild type, a phenotype also restored by XccΔCxanB (Fig. 3), suggesting that the *xanB* gene is involved in this crucial characteristic of the infectious process. In *Stenotrophomonas maltophilia*, *xanB* mutation using a transposon impaired bacterial motility, which was attributed to the lack of the polar flagellum in the mutant cells (44). Therefore, the decreased motility of XccΔxanB may have contributed to its loss of pathogenicity. As is known, phytopathogens use motility mechanisms to reach nutrient-rich surfaces and plant tissues (45). Xcc motility is conferred by a single polar flagellum that allows the bacterium to glide in the epiphytic environment and in the apoplast (46). The disruption of the *wxacO* gene, which participates in LPS synthesis, had a negative impact on Xcc (47). Thus, although it is necessary to confirm that XccΔxanB is defective in LPS production and polar flagellum formation, it is possible that the reduction in motility is related to these two components acting in the pathogenesis of citrus canker.

Biofilm formation is a common ability of bacteria to aggregate in matrices, allowing adhesion to surfaces and providing a dynamic way of life for bacterial populations (48). Although the XccΔxanB strain showed higher biofilm formation than the wild type and XccΔCxanB in the first 24 h, after 48 and 72 h, the pattern was reversed (Fig. 4). This unexpected behavior in the first hours was also observed for *Photorhabdus luminescens*, where PMI-GMP was related only to the biofilm maturation stage and not to the initial stage of its development (48).

A large-scale mutagenesis approach using transposons identified 92 genes related to biofilm formation in Xcc, including *xanB*, which supports the data obtained here using a different methodology to mutate the target gene (49). The formation and maturation process of biofilms involves the participation of exopolysaccharide (EPS) and LPS, polymeric substances that act in cell cohesion and intercellular communication (50). Specifically for Xcc, motility and adhesion are necessary characteristics for the early stages of biofilm formation, while biofilm maturation depends on LPS and EPS, such as xanthan gum (47, 49). Therefore, the participation of the *xanB* gene in the LPS and EPS biosynthesis pathways can also explain the loss of pathogenicity of XccΔxanB. Furthermore, biofilm formation promotes the epiphytic survival of Xcc (51), protects against environmental stresses, such as resistance to ultraviolet radiation, and also acts in defense against host response mechanisms (52). Thus, the reduction in biofilm formation presented by XccΔxanB may explain the *in vivo* results.

XccΔxanB showed threefold lower survival to UV compared to the wild type, and this phenotype was restored by gene complementation (Fig. 5). The increased susceptibility of XccΔxanB to ultraviolet radiation can be explained by the role of XanB in the xanthan biosynthesis pathway, as this EPS has been associated with resistance to various environmental stresses, including exposure to UV radiation (53).

The XanB enzyme is bifunctional, as previously described. *In silico* selected compounds inhibited its pyrophosphorylase activity, which affects the formation of GDP-mannose from mannose-1-phosphate and GTP (54). GDP-mannose is one of the components necessary for the formation of xanthan gum (55), which is a survival mechanism for bacteria as it helps to protect against ultraviolet radiation, freezing, and desiccation. The role of the XanB enzyme on the bacterial surface is still unknown. Although many proteins are involved in Xcc pathogenicity and complex secretion systems have been described as important for successful colonization of the citrus host, this study showed that the absence of a single protein, XanB, was sufficient to cause the loss of bacterial infectivity, evidencing its high potential as a target of biotechnological interest.

The three compounds interact with the amino acids Gly155, Glu173, and Asn192, showing that these residues play a fundamental role in inhibiting the GDP-mannose pyrophosphorylase activity, for which they were specifically designed. Inhibitor i3 does not interact with Asp249, unlike i1 and i2. However, this interaction does not appear to be relevant for the inhibition process, as there was no statistical difference between the three compounds. The inhibitors were specifically designed to target the pyrophosphorylase activity of the enzyme at its active site, not for the isomerase site, as mentioned earlier.

This study opens up future perspectives with broader implications, starting from the premise that XanB is either a target of biotechnological interest in Xcc and is absent in the genome sequences of *Citrus* hosts at NCBI, particularly *C. sinensis* (orange), *C. delicious* (tangerine), and *C. limon* (lemon). Moreover, annotated genomes of over 20 species belonging to the genus *Xanthomonas*, which are responsible for causing several diseases in economically important crops, show the presence of XanB homologs sharing 95% identity or more with XanB of Xcc. Finally, the possibility of molecular optimization of the inhibitors also suggests a promising way forward for future investigation.

## MATERIALS AND METHODS

Detailed descriptions are given in Supplemental Text.

### Bacterial strains, culture media and conditions, and general procedures

Cultures of Xcc and *E. coli* strains, DH5α and BL21 (DE3), were grown on LB agar (Sigma-Aldrich) or LB broth (Sigma-Aldrich), under incubation at 30°C for Xcc and 37°C for *E. coli* strains, and 250 rpm. For the biofilm formation assay, cultures were grown in XAM-M medium (56). Molecular biology procedures were carried out following Ausubel’s protocols (57) with modifications when necessary.

### Construction of *xanB* deleted mutant and complemented strain

The mutant strain was generated via double homologous recombination between *xanB* flanking regions cloned in the pNPTS138 plasmid and the corresponding regions of Xcc genome as standardized by our research group (33, 58, 59). To construct the deletion plasmid, 1-kb regions flanking the ORF XAC3580 were amplified by PCR using Xcc genomic DNA as template and the oligonucleotides specific for the upstream and downstream fragments (Table S1). The deletion plasmid pNPTS138_*xanB* was utilized to transform Xcc by electroporation (60), and the kanamycin-resistant colonies were grown repeatedly in LB and plated on sucrose 10%. An Xcc mutant colony (XccΔxanB) was confirmed by PCR using the *ko-F* and *ko-R* oligonucleotides (Table S1).

A complemented strain was also obtained by double homologous recombination. The PCR-amplified *xanB* gene was cloned into the *Eco*RI unique site of pNPTS138_*xanB* deletion vector (between the two flanking fragments), producing the complementation vector pNPTS138_C*xanB*, which was used in the XccΔxanB mutant transformation to obtain the complemented strain XccΔCxanB.

### Pathogenicity tests in *Citrus* spp. for mutant and complemented strains

The pathogenicity of Xcc, XccΔxanB, and XccΔCxanB strains was evaluated in *Citrus aurantifolia* plants by infiltration and spraying methods. For both assays, isolated colonies were initially cultured in 5 mL of LB broth until reaching an optical density (OD_595 nm_) of 0.4. Subsequently, 100 µL of these cultures was centrifuged at 12,000 × *g* for 15 minutes at 4°C. The resulting pellets were resuspended in 10 mL of 0.9% saline solution, yielding a bacterial suspension of approximately 10^6^ colony-forming unit (CFU)/mL.

In infiltration assays, 150 µL of the bacterial suspensions (or 0.9% saline solution for the negative control) was injected into the abaxial side of the leaves using a 5-mL syringe. To ensure comprehensive assessment, four leaves from independent branches were infiltrated for each condition, with all plants receiving both bacterial and saline solution treatments (23).

For the spraying assay, 10 mL of the bacterial suspensions (or 0.9% saline solution for the negative control) was applied onto quadruplicate plants. The most susceptible leaves were marked at the outset to facilitate consistent application and assessment.

Both infiltration and spraying assays were conducted on two separate days, employing two biological replicates (independent cultures) each time and four experimental replicates (four leaves for infiltration and four plants for spraying) for each bacterial line. The leaves and plants were photographically documented after 20 days (for infiltration) and 28 days (for spraying), allowing for a visual comparison of the symptoms associated with the infectious process.

### Motility, biofilm, and UV resistance assays for *xanB* deletion mutant and complemented strain

Swarming motility of the Xcc, XccΔxanB, and XccΔCxanB strains was assessed following the protocol previously described (61). Bacterial cells were inoculated in the center of the petri dishes containing LB solid medium. The plates were incubated at 30°C for 48 h without shaking. Colony diameter was measured and statistical analysis (Tukey’s test) was performed.

Biofilm formation by XccΔxanB and XccΔCxanB strains was carried out in 96-well plates containing XAM-M medium by incubation at 30°C without shaking for 24, 48, and 72 h. After staining with crystal violet and washes, residual dye from the adhered cells was solubilized and quantified.

Resistance to UV radiation was carried out by exposing Xcc, XccΔxanB, and XccΔCxanB cells to UV radiation from a biological safety cabinet (47). The Xcc, XccΔxanB, and XccΔCxanB strains were cultured in 5 mL of LB at 37°C for 16 h. Subsequently, the optical density at 595 nm was adjusted to 0.1 (approximately 3.10^7^ CFU/mL). In triplicate, 100 µL of these cultures contained in Eppendorf tubes was exposed to a 15-W lamp that produces UV light with a spectrum predominantly peaking at 253.7 nm at a distance of 60 cm from the light source. After 15 minutes of exposure, serial dilutions were performed, and the samples were plated on LB agar for CFU counting and calculation of the survival percentage. Statistical analysis of the data was performed using Tukey’s test.

### *xanB* gene cloning, recombinant expression, and purification

The *xanB* coding sequence (ORF XAC3580) was PCR-amplified using genomic DNA from Xcc and cloned into the pJET v.1.2 vector and subsequently into the pET41a vector (Novagen). After transformation of *E. coli* DH5α and plating, the plasmid from a kanamycin-resistant clone (pET41a_*xanB*) was used to transform *E. coli* BL21 (DE3). A transformant was grown in LB containing kanamycin, and 0.1-mM IPTG was added. After cell sonication, the protein (recombinant XanB) was purified from the soluble fraction by immobilized-metal affinity chromatography (IMAC) and elution with glutathione. After analysis by SDS-PAGE (62), the last eluate fraction was dialyzed and quantified by the UV absorption method.

### XanB homology modeling and virtual screening for inhibitors

#### Construction, evaluation, and validation of the model

The models were generated following the traditional steps of a homology modeling procedure. BLASTp search and selection of homologous structures containing crystallographic data (63) were used as templates. Sequences of these crystallographic structures were aligned with the query protein by using Clustal Omega (25). Models were generated for the query protein using MODELLER (64), and validation was performed by selecting the best model proposal using WHAT IF and SAVES v.5 suite (65).

#### Library preparation

Several commercially available databases for this study, including ZINC (subcollections Natural Stock, Drug-database, and Drug-like) (66, 67), BindingDB subdivision Drugs FDA (68), ChemBridge (with subcollections such as DIVERSet-EXPEXPRESS-Pick Collection and DIVERSet Core Library) (69), Maybridge subdivision Screeening Collection (70), Princeton and IBS (subdivision Natural e Synthetic), which collectively contain approximately 20 million marketable compounds. The compounds were prepared using OMEGA software (71) for conformers generation, with only one conformation per molecule, a strain energy of 9 kcal/mol, and a 0.6-Å cutoff for conformer differentiation (72).

#### Ligand preparation

For our ligand-based virtual screening (LBVS) studies we used the G1P ligand, retrieved from the PDB ID 1H5R complex structure, in its supposed bioactive conformation used here as a query/template for the virtual screening studies.

#### Virtual screening studies

LBVS was the first virtual screening here performed. We use the ROCS (73) and EON (74) software to carry out three-dimensional similarity studies (of shape and electrostatic potential, respectively). After LBVS, SBVS studies were then performed and, thus, the resulting compounds were screened according to the scores obtained in the docking simulations performed using the GOLD software.

Molecules were evaluated regarding their ADME/Tox properties, using the QikProp (75) (from the Schroedinger company) and DEREK (76) (from the Lhasa company) software, respectively.

The “surviving” compounds to the ADME/Tox filter were then submitted to pharmacokinetic analyses performed using QikProp and toxicological analysis performed using DEREK. Finally, for final selection of the compounds, a visual inspection of the interaction/binding modes of these compounds inside the XanB catalytic site was performed.

### XanB enzymatic activities and *in vitro* inhibition by the *in silico* selected compounds

The PMI activity of the recombinant XanB was first assessed by Seliwanoff’s test (77, 78). Briefly, 30 µg of recombinant XanB, D-mannose-6-phosphate at 0.5 mM, and Seliwanoff reagent were added, and results were photographed. The enzyme was replaced by the buffer for the negative control, and fructose-6-phosphate was used instead of D-mannose-6-phosphate at the same concentration of 0.5 mM for the positive control. Tests were performed in triplicate. A second method involving the coupling of three enzymatic reactions (79) was used by quantifying NAPDH formation at 340 nm. The reaction consisted of recombinant XanB 4 ng/µL, mannose-6-phosphate 10 mM, PGI 0.06 U/µL, G6PD 0.06 U/µL, NADP +40 mM, and MgCl_2_ 500 mM (80). The negative control replaced XanB by buffer, while the positive control had fructose-6-phosphate 10 mM instead of mannose-6-phosphate. Triplicate reactions were monitored at 340 nm every 30 seconds. Mean absorbance and standard deviation were calculated. Inhibitors were tested at 1 mM using the same conditions.

GDP-mannose pyrophosphorylase activity of XanB was tested by the method of Davis et al. (81) by conversion of mannose-1-phosphate and GTP into GDP-mannose and pyrophosphate, coupled with pyrophosphatase, which converts pyrophosphate into inorganic phosphate (absorption at 650 nm). Briefly, reaction was composed of XanB 20 ng/µL, D-mannose-1-phosphate 1 mM, GTP 1 mM, MgCl_2_ 500 mM, pyrophosphatase 0.01 U/µL, and dithiothreitol (DTT) 100 mM. For the negative control, XanB was replaced by buffer. Reactions were performed in triplicate, and pyrophosphate detection was performed using a reagent containing malachite green, ammonium molybdate, and Triton in HCl. Absorbance at 650 nm was measured, and the mean absorbance and standard deviation were calculated. For the inhibition tests, the same conditions were repeated, but XanB was incubated with inhibitors at 1 mM for 30 minutes at 30°C.

### *In vivo* pathogenicity assays of Xcc in *Citrus* spp. in presence of XanB inhibitors

The protective effect of XanB inhibitors was assessed in *C. aurantifolia* and *C. sinensis*. Each treatment consisted of three plants, with 10 leaves per plant. Inhibitors were sprayed at a concentration of 1 mM on the adaxial and abaxial surfaces of the leaves. In the assay with *C. sinensis*, copper was applied at 1.08 g/L, which was the same as the field application rate (3). Twenty-four hours after inhibitor application, the plants were spray-inoculated with a Xcc suspension on both surfaces of the leaves and placed in a humid chamber for 24 h. Evaluation of the 10 leaves of each plant was conducted using ImageJ software.

## Data Availability

All data are available in the paper or in the supplemental material.

## SUPPLEMENTAL MATERIAL

Supplemental material spectrum.03673-23-s0001.docx Additional experimental details, Figures S1 to S7, and Table S1.

Supplemental text spectrum.03673-23-s0002.docx Additional text for Material and Methods.

ASM does not own the copyrights to Supplemental Material that may be linked to, or accessed through, an article. The authors have granted ASM a non-exclusive, world-wide license to publish the Supplemental Material files. Please contact the corresponding author directly for reuse.
