# Supplementary material for: GDP-mannose pyrophosphorylase is an efficient target in Xanthomonas citri for citrus canker control
Source: Microbiol Spectr. 2024 May 9;12(6):e03673-23. doi: 10.1128/spectrum.03673-23 (PMC11237706; doi:10.1128/spectrum.03673-23)
Supplement: Supplemental material — Additional experimental details, Figures S1 to S7, and Table S1. [file spectrum.03673-23-s0001.docx]

**SUPPLEMENTARY INFORMATION:**

Xcc XanB model presented a total score on WHAT IF of - 0.782. Also, all the scores ​​obtained using PROCHECK were within the expected values ​​for 1.9 Å resolution protein structures (referring to the PDB ID 1H5R structure, used as template for the homology modeling procedure). In addition, tetrahedral distortion of alpha carbons of the final model had lower values than the crystallographic one. The Ramachandran plot obtained for such model did not indicate any unfavorable phi and psi angles for the amino acid residues, especially in the interaction site region, with 91.6% of the residues located into “highly favorable” regions. Although the residue Asn88 could be located in a “generously permitted” region, its torsion angles were “inherited” from the crystallographic structures here used as templates.

Analysis performed using the Verify-3D, ERRAT and PROVE methodologies indicated positive results for the model. Using Verify-3D, 80% of the amino acids scored 0.2 or higher on the 3D-1D profile, and using ERRAT, a high-quality factor of 85.502 was obtained. Finally, using PROVE, both the Z-score and the Z-score RMS obtained were above of the expected for structures with high crystallographic resolution, where the Z-score had an average of 0.274 and the Z-score RMS had a value of 1.539.In the case of the human PMI model, BLASTp identified two homologue structures (PDBs IDs 5NW7 and 3H1M), with sequence identities of 35% and 40%, respectively, and the referred sequence alignments were performed using Clustal Omega.

The final model thus obtained has a WHAT IF score of - 0.675 and, according to PROCHECK, all the values are within the expected range for 1.85 Å resolution protein structures (referring to the PDB ID 5NW7 structure). The tetrahedral distortion of alpha carbons of the model has a lower value than the crystallographic one (below 2.5). The Ramachandran plot showed 89.2% of its residues in “highly favorable” regions, whereas only 0.8% are located in “forbidden” regions: Ala379, Asn91 and Ser184, which are not located into the PMI active site.

Finally, using VERIFY-3D, 95.5% of the amino acid residues have a score equal to or greater than 0.2 in the 3D-1D profile, whereas using ERRAT a quality factor of 61.6867 was obtained, and using PROVE the Z-score and the RMS Z-score were above the region of interest, where the Z-score has an average value of 0.274 and the Z-score RMS with a value of 1.539.

In the case of the human PMI model, BLASTp identified two homologue structures (PDBs IDs 5NW7 and 3H1M), with sequence identities of 35% and 40%, respectively, and the referred sequence alignments were performed using Clustal Omega.

The final model thus obtained has a WHAT IF score of - 0.675 and, according to PROCHECK, all the values are within the expected range for 1.85 Å resolution protein structures (referring to the PDB ID 5NW7 structure). The tetrahedral distortion of alpha carbons of the model has a lower value than the crystallographic one (below 2.5). The Ramachandran plot showed 89.2% of its residues in “highly favorable” regions, whereas only 0.8% are located in “forbidden” regions: Ala379, Asn91 and Ser184, which are not located into the PMI active site.

Finally, using VERIFY-3D, 95.5% of the amino acid residues have a score equal to or greater than 0.2 in the 3D-1D profile, whereas using ERRAT a quality factor of 61.6867 was obtained, and using PROVE the Z-score and the RMS Z-score were above the region of interest, where the Z-score has an average value of 0.274 and the Z-score RMS with a value of 1.539.

An important part of the data obtained from the ROC curve is a standard measure called the “area under the curve” (AUC), a number that ranges from 0 to 1 and shows the overall performance of the study, that is, the probability of such model to classify a positive part of the data as negative and/or vice versa. The AUC ROC value obtained for this study with our model was 0.760, close to 1 (ideal value).

In a ROC curve, the diagonal line on the graph corresponds to experiments in which the scoring function would not be discriminated. Another part of the data obtained using the web server Screening Explorer (at http://stats.drugdesign.fr/) is the forecast curve that allows detecting whether the variations in the activity probability are important enough to induce the selection of a threshold for virtual screenings ^1^.

For Xcc, this curve is above the limit line, making it a good result and showing that the model was able to discriminate between actives from inactives regarding the compounds here used. Associated with this data is the standardized total gain (TG) ^2^, which summarizes the discrimination of active compounds in respect to the variation of scores across the data set of molecules ^1^.

The TG value variates from 0 to 1, and its analysis must be associated with the value obtained for AUC. For XanB of Xcc, the TG value obtained was 0.312, which, associated with an AUC ROC of 0.760, reveals that the methodology here used was able to discriminate the active from the inactive compounds for such protein model.

Finally, a final part of the data obtained is the “enrichment curve”, which allow us evaluating the early recovery of active compounds selected using virtual screening methodologies. The curve for our study is again above the threshold line as well as together with the enrichment factor (EF), which corresponds to the fraction of active compounds obtained when a certain percentage of inactive compounds is found, and it reveals that for ten recovered inactive compounds, only two of them are active.

For human PMI, the forecast curve obtained for such model is also above the limit line and with a TG of 0.444, which, associated with an AUC ROC of 0.792, reveals that such methodology here used had a good performance and it could be reproducible under similar experimental conditions. Finally, we obtained a prediction curve that again is above the threshold line and, together with an EF of 2.31, it shows that for ten recovered inactive compounds, two of them are active. All the ROC curves can be visualized in Figure 8.

| (A) 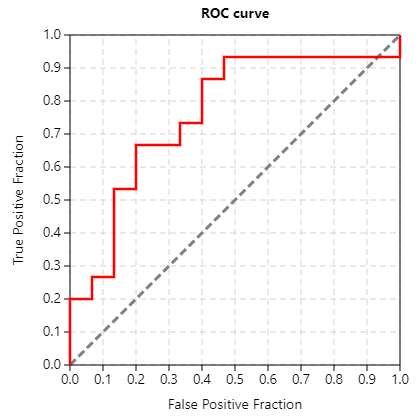 | (B) 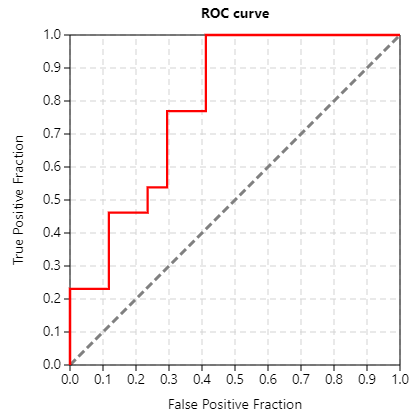 |
| --- | --- |
| **Fig. 1 ROC curves obtained for the study of the 30 compounds reported as more and less potent PMI inhibitors**, according to the BindingDB web server, generated for the Xcc XanB model (A) and the Human PMI model (B) as well. | |

**REFERENCES OF SUPPLEMENTARY MATERIAL:**

1. Empereur-Mot, C. *et al.* Predictiveness curves in virtual screening. *J. Cheminform.* **7**, 1–17 (2015).

2. Bura, E. & Gastwirth, J. L. The Binary Regression Quantile Plot: Assessing the Importance of Predictors in Binary Regression Visually. *Biometrical J.* **43**, 5–21 (2001).

**SUPPLEMENTARY FIGURES AND TABLES:**


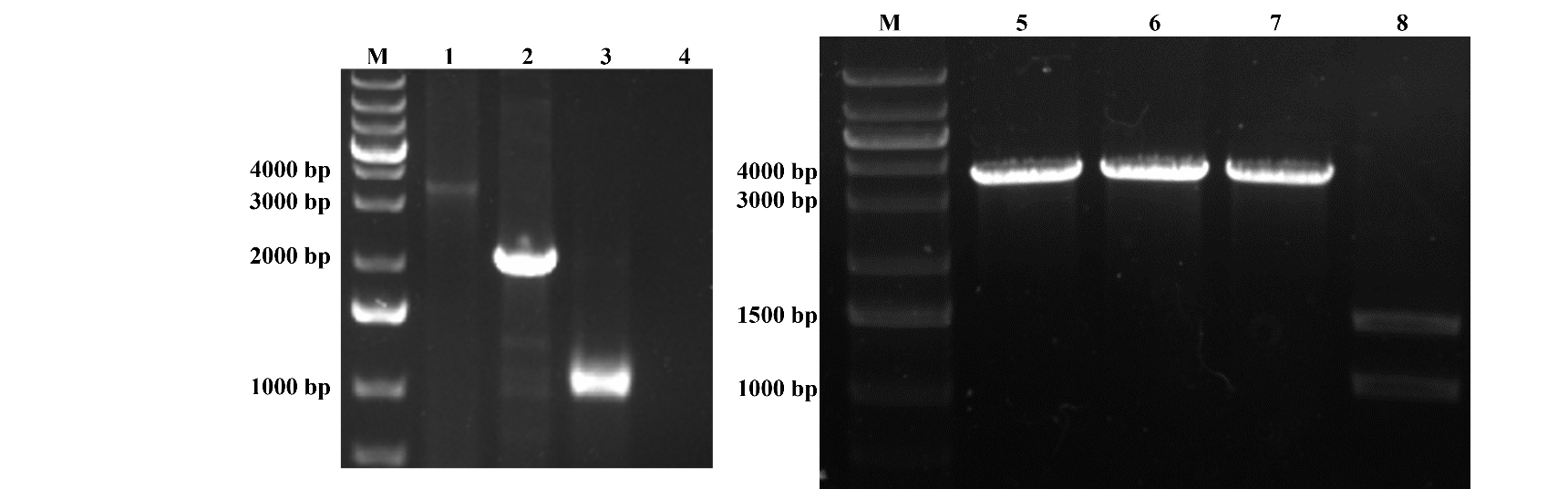


**Fig. S1 Confirmation of *xanB* gene deletion and gene complementation.** On the left, analysis of PCR products resulting from reactions using wild-type and mutant Xcc colonies as templates, as well as the pNPTS138_xanB deletion vector. All reactions used oligonucleotides that hybridize to 50 bp from each one of the two 1 kb flanking fragments. **M** **|** GeneRuler Molecular Size Standard 1 kb Plus DNA Ladder (Fermentas); **1 |** PCR product using Xcc colony as template. **2 |** PCR product using XccΔxanB colony as template. **3 |** PCR product using XccΔxanB as template, digested with *Eco*RI endonuclease. **4 |** PCR product using pNPTS138_xanB vector as template. Relevant bands of the molecular size pattern are indicated. On the right, analysis of PCR products resulting from reactions using colonies of Xcc and complemented strain (XccΔCxanB) as templates. **5 |** PCR product using wild-type Xcc as template. **6 |** PCR product using wild-type Xcc as template, digested with *Eco*RI enzyme. **7 |** PCR product using XccΔCxanB as template. **8 |** PCR product using XccΔCxanB as template, digested with *Eco*RI enzyme. Relevant bands of the molecular size pattern are indicated.


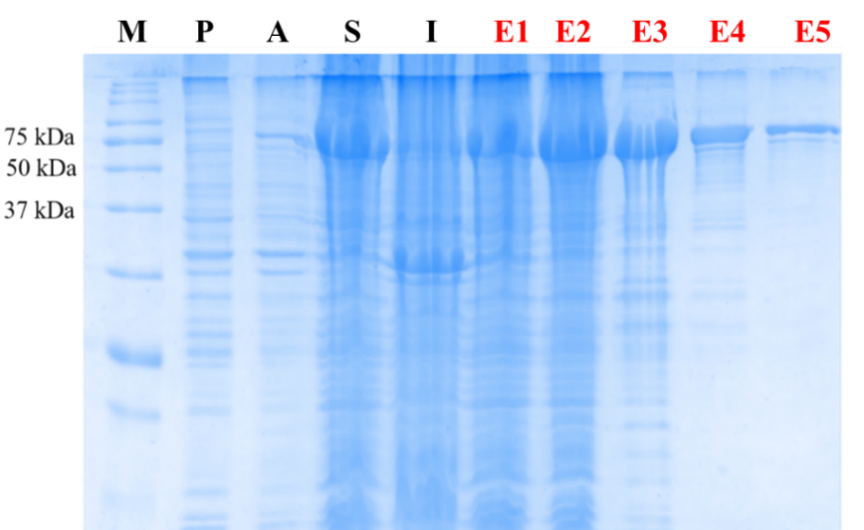


**Fig. S2 Analysis of expression, solubility and purification of the recombinant XanB by SDS-PAGE.** Recombinant XanB (approximately 85 kDa) was purified in immobilized glutathione column, after heterologous expression using the vector pET41a_xanB. **M** | Precision Plus Protein™ Dual Color (Bio-Rad) molecular mass standard. **P** | Prior IPTG induction. **A** | After induction by IPTG. **S** | Soluble fraction of the lysate. **I** | Insoluble fraction of the lysate. The protein was eluted by applying 5 column volumes (5 ml) of 10 mM reduced glutathione, with **E1-E5** (shown in red) being the eluates for each of the applied volumes (1 ml). The expression assay resulted in a yield of 6 mg of recombinant protein per liter of culture medium.


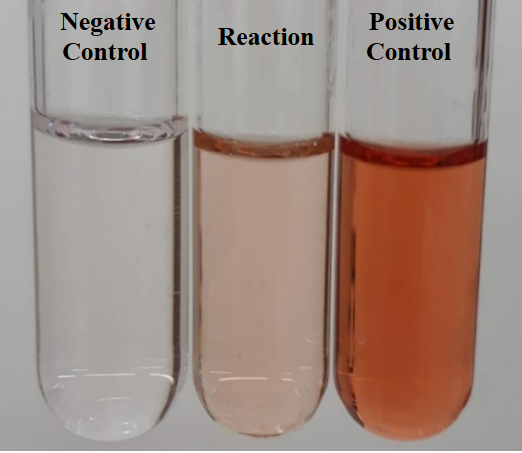


**Fig. S3 Isomerase activity of the recombinant XanB by the Seliwanoff´s test.** **Negative control |** Composed by 0.1 M D-mannose-6-phosphate, buffer and Seliwanoff's reagent. **Reaction |** Composed by 0.1 M D-mannose-6-phosphate, recombinant XanB (300 ng/μl) in buffer and Seliwanoff's reagent. **Positive Control |** Composed by 0.1 M compound of D-fructose-6-phosphate, buffer and Seliwanoff's reagent. The red-brown color in the “Reaction” tube allowed the conclusion that the recombinant XanB also has the predicted catalytic activity of converting D-mannose-6-phosphate to D-fructose-6-phosphate. All buffers used consisted of 50 mM Tris-HCl pH 8.0 100 mM NaCl.


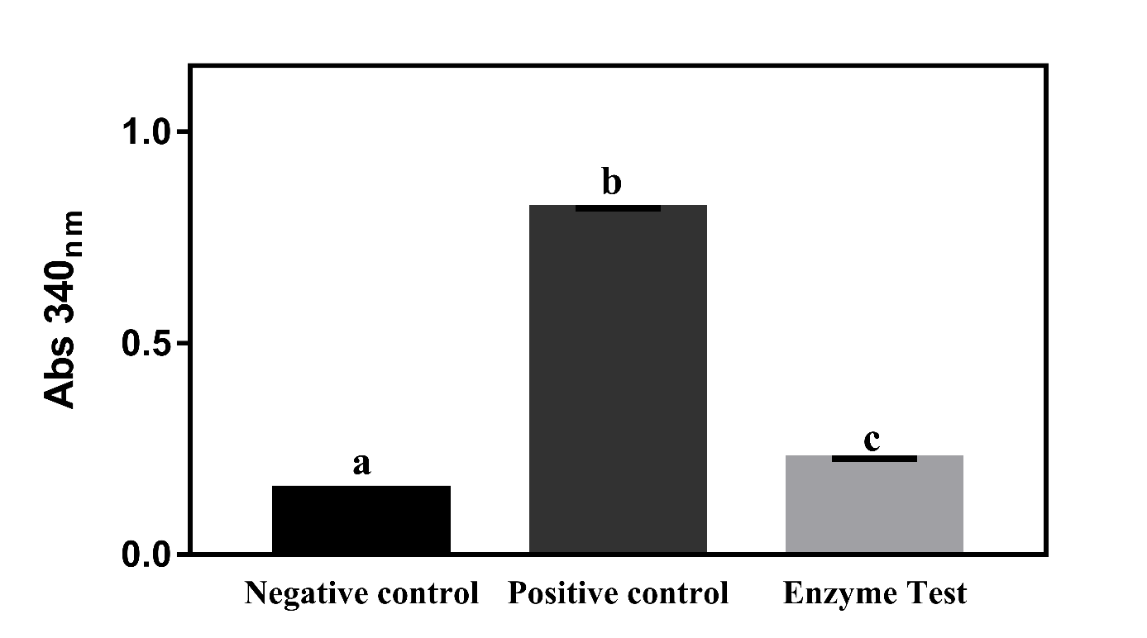


**Fig. S4 Isomerase activity of the recombinant XanB by a coupling method with PGI and G6PD. Negative control |** Composed by D-mannose-6-phosphate (10 mM), PGI (0,06 U/μl), G6PD (0,06 U/μl), NADP+ (40 mM), MgCl_2_ (500 mM) and buffer**. Positive Control |** The same composition as the Negative control, with the replacement of the D-mannose-6-phosphate by D-fructose-6-phosphate**. Enzyme Test |** The same composition as the Negative control, with the replacement of the buffer by the recombinant XanB (4 ng/μl). All buffers used consisted of 50 mM Tris-HCl pH 8.0 100 mM NaCl. Error bars indicate the absolute standard deviation of each of the triplicates. Columns followed by the same letter do not show significant difference using Tukey´s test (*P=* 0.05).

**
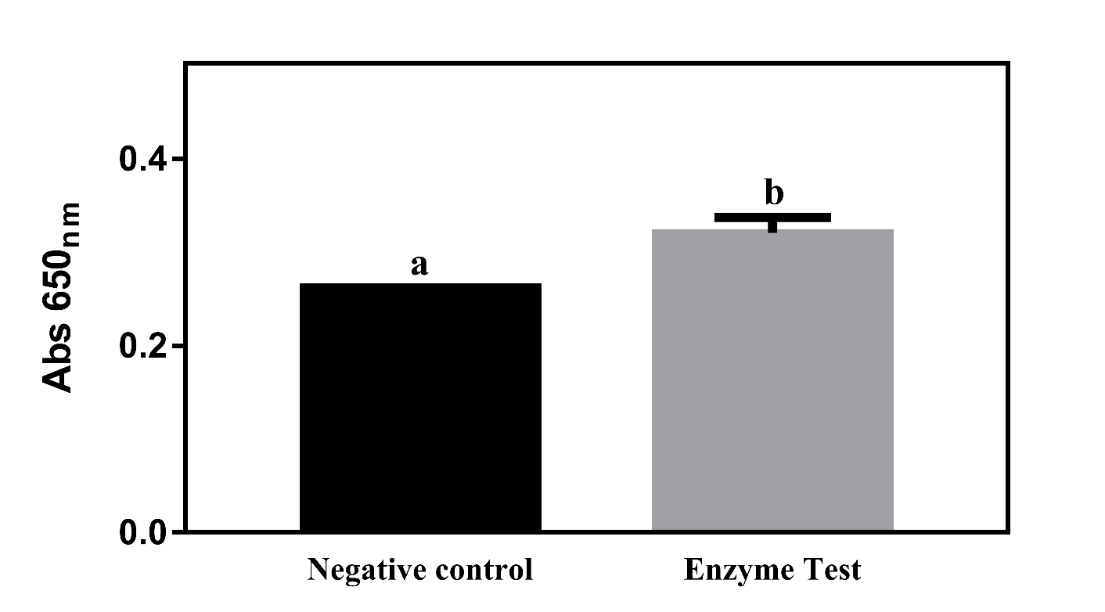
**

**Fig. S5 Pyrophosphorylase activity of the recombinant XanB by the pyrophosphatase coupling method. Negative control |** Composed by D-mannose-1-phosphate (1 mM), GTP (1 mM), MgCl_2_ (500 mM), pyrophosphatase (0.01 U/µl), DTT (100 mM) and buffer 50 mM Tris-HCl pH 8.0 100 mM NaCl**. Enzyme Test |** The same composition as the Negative control, with the replacement of buffer by recombinant XanB (20 ng/μl)**.** Error bars indicate the absolute standard deviation of each of the triplicates. Columns followed by the same letter do not show significant difference using Tukey´s test (*P=* 0.05).

| **** |
| --- |
| **Fig. S6 Multiple alignment analysis obtained for XanB after refinement.** Sequences and the respective crystallographic structures indicated as homologous by BLASTp (PDB ID 1H5R, 2CU2, 2X5S and 2QH5), were used as templates. |
| *Amino acids with a dark blue color belong to the β-sheet, while those with a green color belong to the α-helices. Amino acids that have had their rotamers modified are highlighted in blue. The asterisk (*) symbology represents the presence of a single fully conserved residue, a colon (:) indicates conservation between groups of strongly similar properties, and a period (.) indicates conservation between groups of weakly similar properties.* |


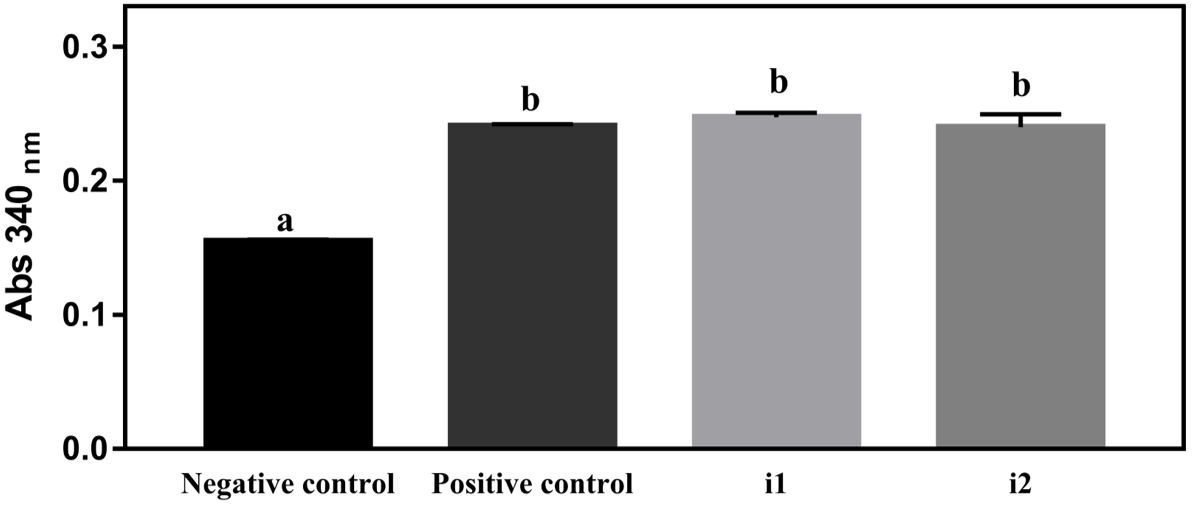


**Fig. S7 Evaluation of the potential to inhibit isomerase activity for i1 and i2. Negative control |** Reaction composed by mannose-6-phosphate (10 mM), PGI (0,06 U/μl), G6PD (0,06 U/μl), NADP+ (40 mM), MgCl_2_ (500 mM) and buffer**. Positive Control |** The same composition as the Negative control, with the replacement of the buffer by the recombinant XanB (4 ng/μl). **i1** and **i2 |** The same composition as the Positive control, with the addition of i1 or i2 at 1 mM. All buffers used consisted of 50 mM Tris-HCl pH 8.0 100 mM NaCl. Error bars indicate the absolute standard deviation of each of the triplicates. Statistical analysis was performed using the Tukey´s test, with a confidence level of 95%.

**Table S1.** Primers utilized for *xanB* deletion, complementation and heterologous expression. Underlined sequences in the nucleotide sequences indicate the restriction sites.

| **Primer ID** | **Target gene or region** | **Sequences (5´->3´)** | **Application in this study** | **Restriction enzymes** |
| --- | --- | --- | --- | --- |
| *up-F* | 1 kb upstream to the ORF XAC3580 | TATATAAAGCTTATACCGCAGCGCCAGGC | Deletion vector (pNPTS138_xanB) | *Hind*III |
| *up-R* | 1 kb upstream to the ORF XAC3580 | CGTATAGAATTCGGGCGGGGGAAACTC | Deletion vector (pNPTS138_xanB) | *Eco*RI |
| *down-F* | 1 kb downstream to the ORF XAC3580 | TTAACAGAATTCTCGCGCGTCTCAACGTAG | Deletion vector (pNPTS138_xanB) | *Eco*RI |
| *down-R* | 1 kb downstream to the ORF XAC3580 | ATAATTGCTAGCTTGTACGACAACCTGTACCC | Deletion vector (pNPTS138_xanB) | *Nhe*I |
| *ko-F* | 50 bp from the 1 kb upstream region | GATCAGCTCGGACACCGG | Confirmation of deletion or complementation |  |
| *ko-R* | 50 bp from the 1 kb downstream region | GGACCGACAACATCATCTTCTCG | Confirmation of deletion or complementation |  |
| *comp-F* | ORF XAC3580 | TATATAGAATTCATGAGCGACGTCCTACCCAT | Complementation vector (pNPTS138_CxanB) | *Eco*RI |
| *comp-R* | ORF XAC3580 | TATATAGAATTCTTAGGCGCGGCCGTAGG | Complementation vector (pNPTS138_CxanB) | *Eco*RI |
| *exp-F* | ORF XAC3580 | TATATAGAATTCATGAGCGACGTCCTACCCAT | Expression vector (pET41a_xanB) | *Eco*RI |
| *exp-R* | ORF XAC3580 | TATATACTCGAGTTAGGCGCGGCCGTA | Expression vector (pET41a_xanB) | *Xho*I |
