# Supplementary material for: GDP-mannose pyrophosphorylase is an efficient target in Xanthomonas citri for citrus canker control
Source: Microbiol Spectr. 2024 May 9;12(6):e03673-23. doi: 10.1128/spectrum.03673-23 (PMC11237706; doi:10.1128/spectrum.03673-23)
Supplement: Supplemental text — Additional text for Material and Methods. [file spectrum.03673-23-s0002.docx]

**SUPPORTING INFORMATION**

**SI Text -Material and Methods**

**Bacterial strains, culture media and conditions, and general procedures.** *Xanthomonas citri* subsp*. citri* strain 306 (Xcc) was kindly provided by Fundo de Defesa da Citricultura (Fundecitrus, Araraquara, SP, Brazil). Bacteria were stored at -80°C in LB broth (Sigma-Aldrich) for *X. citri* and *E. coli* strains, with 10% glycerol. XAM-M medium, a pathogenicity-inducing medium for Xcc, was composed of 7.57 mM (NH_4_)_2_SO_4_, 33.06 mM KH_2_PO_4_, 60.28 mM K_2_HPO_4_, 1.7 mM sodium citrate (C_6_H_5_Na_3_O_7_.2H_2_O), 1 mM MgSO_4_, 0.03% (w/v) casamino acids, 10 mM fructose, 10 mM sucrose and 1 mg/mL bovine serum albumin (BSA) (Sigma-Aldrich), and adjusted to pH 5.4. The pNPTS138 suicide vector (Alley, unpublished results) was kindly provided by Prof. Dr. Henrique Ferreira from UNESP (Rio Claro, SP, Brazil) and used for *xanB* deletion and complementation.

**Construction of *xanB* deleted mutant and complemented strain**

To construct the deletion plasmid, 1 kb regions flanking the ORF XAC3580 were obtained from the Xcc strain 306 genome at NCBI, and were amplified by PCR using Xcc genomic DNA as template and the oligonucleotides *up-F* and *up-R* for the upstream fragment, *down-F* and *down-R* for the downstream fragment (Table S1).

The PCR reaction started with a denaturation step at 98°C for 10 minutes, followed by 35 cycles at 98°C for 30 seconds, 62°C for 30 seconds, and 72°C for 1 minute, and a final extension step at 72°C for 10 minutes. The resulting PCR products were cloned into the pJET 1.2 vector (Thermo Scientific™) and sequenced^1^. Next, the two flanking regions for the *xanB* gene were excised from each pJET 1.2 vector using the respective enzyme pairs and cloned *in tandem* into the pNPTS138 vector between the *Hind*III and *Nhe*I sites. To do this, the pNPTS138 vector was digested with enzymes compatible with one of the flanking regions ends, fragment-ligated, and transformed into *E. coli* DH5α. The recombinant plasmid obtained was then digested with enzymes compatible with the second flanking region, and ligation and *E. coli* transformation were performed to obtain the deletion plasmid^2,3^, which was named pNPTS138_xanB. After Xcc eletroctroporation, transformed bacteria were plated on LB agar supplemented with kanamycin (30 μg.ml^-1^), as described previously^2,3^. The colonies were grown repeatedly overnight in LB medium without kanamycin, and then plated on LB agar containing sucrose 10%. Only the bacteria that had the plasmid either excised from the genome together with *xanB* gene, and cured, could grow on this medium, while cells resulting from single recombination have the plasmid integrated into the genome and are also eliminated. To confirm the Xcc mutant colonies (XccΔxanB), PCR was performed using the *ko-F* and *ko-R* oligonucleotides (Table S1), which hybridize 50 bp from each of the two 1 kb flanking fragments.

A complemented strain was obtained by double homologous recombination, with the following modifications. The *xanB* gene was cloned into the pNPTS138_xanB vector, between the previously cloned flanking regions of the gene. For this, the *xanB* gene coding region was amplified by PCR using genomic DNA and the *comp-F* and *comp-R* oligonucleotides (Table S1). PCR amplification was performed on a C1000 Touch Thermal Cycler (Bio-Rad), with 50 μl total volume reaction containing 500 ng of Xcc genomic DNA, 2 μM from each primer, 2.5 U of Phusion High Fidelity DNA Polymerase, 1.5 mM MgCl_2_ and 0.8 mM dNTP (0.2 mM each). PCR was initiated with a denaturation step at 98°C for 10 minutes and 35 cycles at 98°C for 30 seconds, 62°C for 30 seconds and 72°C for 1.5 minute, followed by a final extension step at 72°C for 10 minutes. PCR product was purified by agarose gel electrophoresis and cloned into the pJET 1.2 vector (Thermo Scientific™), followed by *E. coli* DH5α transformation and plating on LB agar supplemented with ampicillin (100 µg.ml^-1^). Clones containing the ORF XAC3580 insert (positive clones) were confirmed by digestion with *Eco*RI followed by agarose gel electrophoresis, and also nucleotide sequencing^1^. This gene fragment was then cloned into the *Eco*RI unique site of pNPTS138_xanB deletion vector, located between the two 1 kb flanking fragments, producing the complementation vector pNPTS138_CxanB, whose construction was checked by restriction analysis using 0.8% TAE agarose gel electrophoresis. As the *xanB* gene fragment used in this cloning has an *Eco*RI restriction site at both ends, the right fragment orientation in the pNPTS138_CxanB vector was confirmed by PCR. The complementation vector pNPTS138_CxanB was used in the transformation of the XccΔxanB mutant strain to obtain the complemented strain XccΔCxanB.

**Pathogenicity tests in *Citrus* spp. for *xanB* deletion mutant and complemented strain**

The pathogenicity of Xcc, XccΔxanB and XccΔCxanB strains was evaluated in *Citrus aurantifolia* plants in a greenhouse with controlled temperature and humidity using the spraying and infiltration methods. For both methods, isolated colonies were grown in 5 ml of LB broth until they reached an OD_595 nm_ of 0.4. Next, 100 μl of these cultures were centrifuged at 12,000 x *g* for 15 minutes at 4°C, and the resulting pellets were resuspended in 10 ml of 0.9% saline solution to obtain a suspension containing 10^6^ CFU.ml^- 1^ for each strain. For the infiltration tests, 150 μl of each bacterial culture (or 0.9% saline solution for the negative control) were injected into the abaxial surface of the leaves using a 5-ml syringe. Four leaves from independent branches of the same host plant were infiltrated for each sample and the negative control. For the spraying test, bacterial suspensions containing 10^6^ CFU.ml^- 1^ (or 0.9% saline solution for the negative control) were sprayed on a group of four plants, which had the most susceptible (young) leaves marked at the beginning of the experiment. The infiltration and spraying tests were repeated on two different days, using two biological replicates (independent cultures) each day and four experimental replicates (four leaves for the infiltration test and four plants for the spraying test) for each strain. The leaves were scanned after twenty days (infiltration) and twenty-eight days (spraying) to visually compare the symptoms of the infectious process.

**Motility, biofilm and UV resistance assays for *xanB* deletion mutant and complemented strain**

Swarming motility of the Xcc, XccΔxanB and XccΔCxanB strains was assessed following the protocol previously described^4^. Overnight cultures of the strains were prepared in LB medium at 30ºC with continuous shaking at 200 rpm. The cultures were adjusted to an OD_595 nm_ of 0.3, and 3 µl of each culture were inoculated in the center of the 5.1 cm diameter Petri dishes containing LB medium with 0.7% agar. The plates were incubated at 30ºC for 48 hours without shaking. The plates were then digitally recorded, and the colony diameter was measured using the ImageJ software (https://imagej.nih.gov/ij/). Statistical analysis was performed using Minitab software (version 15.2) with the Tukey´s test at a 95% confidence level.

The measurement of the capacity for biofilm formation of the XccΔxanB and XccΔCxanB strains, in comparison to the wild-type Xcc, was carried out as follows. The strains were grown in 96-well plates containing 150 μl of XAM-M medium, with the OD_595nm_ of the cultures initially adjusted to 0.3. The cultures were incubated at 30°C without shaking for 24, 48 and 72 h. At the end of each time point, the wells were washed three times with 0.9% NaCl, stained with 150 μl of 0.1% crystal violet for thirty minutes, and then washed three times with distilled water. Finally, the residual dye from the adhered cells was solubilized in 150 μl of 70% ethanol and quantified by measuring the absorbance at 595 nm^5^.

The evaluation of resistance to ultraviolet radiation (UV) was carried out by cultivating Xcc, XccΔxanB and XccΔCxanB strains in 5 ml of LB broth at 37 ºC for 16 hours. Then, the OD_595 nm_ was adjusted to 0.1. In triplicate, 100 μl of these cultures contained in Eppendorf tubes were exposed to UV radiation from the biological safety cabinet, at a distance of 60 cm from the light source, as previously described^6^. After 15 minutes of exposure, serial dilution was performed and plated on LB agar for CFU counting. Statistical analysis of the data was performed using the Tukey´s test, with a confidence level of 95%, using Minitab software (15.2).

***xanB* gene cloning, recombinant expression and purification**

The *xanB* coding sequence (ORF XAC3580) was PCR-amplified using genomic DNA from Xcc as template, which was extracted using Wizard® Genomic DNA Purification Kit (Promega), and the *exp-F* and *exp-R* oligonucleotides (Table S1), under the same conditions as those used for the construction of the complementation vector pNPTS138_CxanB. The PCR product was cloned into the pJET 1.2 vector, as described above, and the resulting insert was sequenced. Next, the insert was cloned into the pET41a vector (Novagen) between the same restriction sites used previously. After transformation of *E. coli* DH5α followed by plating on LB agar containing kanamycin (30 µg.ml^-1^), a positive clone was obtained and named pET41a_xanB. This clone was used to transform *E. coli* BL21(DE3) to obtain an IPTG-inducible expression system.

To express the recombinant protein, *E. coli* BL21(DE3) cells transformed with pET41a_xanB were grown in LB broth containing kanamycin (30 µg.ml^-1^) until the OD_595nm_ reached 0.4. At this point, 0.1 mM IPTG was added, and the cells were further incubated on an orbital shaker at 250 rpm for 19 h at 18°C. The cells were harvested by centrifugation (12,000 x *g*, 20 min at 4°C), resuspended in 50 mM Tris-HCl pH 8.0 100 mM NaCl buffer, and sonicated (Sonic Dismembrator 500, Fisher Scientific) under an ice bath for five 30-second ultrasound pulses, with 1-minute interval and 30% amplitude. The lysate was then centrifuged at 12,000 x *g* for 10 minutes at 4ºC to separate the soluble and insoluble cellular fractions. The resulting protein, which had a fused N-terminal GST (glutathione S-transferase) and encodes for PMI (phosphomannose isomerase) and GMP (**GDP-D-mannose pyrophosphorylase**) activities was designated as recombinant XanB.

The recombinant XanB was purified using immobilized metal affinity chromatography (IMAC). The soluble fraction of the cell lysate was added to 0.5 ml Glutathione Sepharose® 4 Fast Flow (GE Healthcare), and the recombinant protein was eluted in five 1-ml aliquots using 5 ml of 10 mM reduced glutathione. The flow-through and eluate aliquots were analyzed by SDS-PAGE^7^, together with the soluble and insoluble cellular fractions, to confirm protein solubility. The last 4-ml eluate fraction was dialyzed against 50 mM Tris-HCl pH 8.0 100 mM NaCl, and quantified by the UV absorption method at 280 nm for use in enzymatic activity assays. To analyze the two enzymatic activities of recombinant XanB, the expression and purification procedures were repeated with modifications, followed by dialysis to obtain the enzyme free from glutathione.

**XanB enzymatic activities and *in vitro* inhibition by the *in silico* selected compounds**

The PMI activity of the recombinant XanB was assessed using two methods. PMI catalyzes the reversible isomerization of D-mannose-6-phosphate to D-fructose-6-phosphate. The Seliwanoff´s test was used to detect the formation of ketosis, with the reagent consisting of resorcinol 0.05% (w/v, final concentration) in hydrochloric acid (HCl) diluted in water (1:1)^8,9^. Reactions included 30 μg of recombinant XanB, D-mannose-6-phosphate (disodium salt, Sigma-Aldrich) at a final concentration of 0.5 mM in a final volume of 500 µl. After 2 hours of incubation, 3 ml of the Seliwanoff reagent were added and the results were photographed. The enzyme volume was replaced by the buffer in which it was solubilized (50 mM Tris-HCl pH 8.0 100 mM NaCl) for the negative control, and fructose-6-phosphate was used instead of D-mannose-6-phosphate at the same concentration of 0.5 mM for the positive control. Tests were performed in triplicate.

To assess the PMI activity of the recombinant XanB, a second method involving the coupling of three enzymatic reactions^10^ was used. The method utilizes two commercially available enzymes – phosphoglucose isomerase (PGI) and glucose-6-phosphate dehydrogenase (G6PD), both purchased from Sigma-Aldrich. The activity was indirectly measured by quantifying the formation of NAPDH in the last coupling reaction, by measuring the absorbance at 340 nm. To perform this assay, a reaction mixture was set up in a final volume of 200 μl, consisting of recombinant XanB (4 ng/μl), mannose-6-phosphate 10 mM (Sigma-Aldrich), PGI (0,06 U/μl, Sigma-Aldrich), G6PD (0,06 U/μl, Sigma-Aldrich), NADP+ (40 mM (Sigma-Aldrich), MgCl_2_ 500 mM (Sigma-Aldrich)^11^. The negative control replaced the recombinant XanB solution with buffer 50 mM Tris-HCl pH 8.0 100 mM NaCl, while the positive control used fructose-6-phosphate 10 mM instead of mannose-6-phosphate 10 mM. Triplicate reactions were incubated at 25ºC, and absorbance measurements were taken at 340 nm every 30 seconds. After two hours, the mean absorbance and standard deviation were calculated for each set of reactions. All inhibitors were tested at the final concentration of 1 mM, using the same conditions as specified above.

The GDP-mannose pyrophosphorylase activity of recombinant XanB was tested following the method of Davis et al. (2004)^12^. This reaction converts the substrates mannose-1-phosphate and GTP into GDP-Mannose and pyrophosphate. The method involves coupling of this reaction with pyrophosphatase, which converts pyrophosphate (PPi) into inorganic phosphate, which absorbs at 650 nm. The reaction was carried out in a final volume of 100 μl, containing recombinant XanB (20 ng/μl), D-mannose-1-phosphate (1 mM, Sigma-Aldrich), GTP (1 mM, Sigma-Aldrich), MgCl_2_ (500 mM), pyrophosphatase (0.01 U/µl, Sigma-Aldrich), and DTT (100 mM, Sigma-Aldrich). The negative control was prepared by replacing the volume of recombinant XanB with buffer 50 mM Tris-HCl pH 8.0 100 mM NaCl. Reactions were performed in triplicate, incubated for 20 minutes at 25ºC, and then 100 μl of inorganic pyrophosphate detection reagent was added, which contained 0.06% (w/v) malachite green (Sigma-Aldrich), 0.4% (w/v) ammonium molybdate (Sigma-Aldrich), 0.1% (v/v) Triton X-100 (Sigma-Aldrich) in HCl 0.7 N. After 5 minutes, the absorbance at 650 nm was measured, and the mean absorbance and standard deviation were calculated. For the inhibition tests, the same conditions were repeated, but recombinant XanB was incubated with inhibitors for 30 minutes at 30ºC (final concentration of 1 mM).

***In vivo* pathogenicity assays of Xcc in *Citrus* spp. in presence of XanB inhibitors**

The protective effect of XanB inhibitors was assessed in *C. aurantifolia* and *C. sinensis*. Each treatment consisted of three plants, with 10 leaves per plant. Inhibitors were sprayed at a concentration of 1 mM on the adaxial and abaxial surfaces of the leaves. In the assay with *C. sinensis*, copper oxychloride (50% metallic copper, Recop, Albaugh) was applied at a concentration of 1.08 g/l, which was the same as the field application rate^3^. Twenty-four hours after inhibitor application, the plants were spray-inoculated with a Xcc suspension at 10^6^ UFC/ml on both surfaces of the leaves, and then placed in a humid chamber for 24 hours. Evaluation of the 10 marked leaves of each plant was conducted at 35 dpi, with the number of lesions on each leaf divided by the leaf area using ImageJ software (<https://imagej.nih.gov/ij/>).

**XanB homology modeling and virtual screening for inhibitors**

Only the molecules containing suitable properties for a potential/promising agrochemical (QPPCaco <25, QPPMDCK <25, Human Oral Absorption = 1 and Percentual Human Oral Absorption < 25%) follows to the next stage, where the potential toxicity of the compounds against the human body was mainly evaluated.

The descriptors used in QikProp were: log CACO2, log MDCK, percentage of oral absorption and quality of the human oral absorption model. All compounds that had log CACO2 and log MDCK values greater than or equal to 500, oral absorption potential greater than or equal to 80%, and quality of the human absorption model equal to high were rejected. In the toxicological analysis performed using DEREK, the rejected compounds were those for which at least one toxicity alert was fired according to the Custom Prediction and Lhasa Prediction as well.

On visual inspection of the compounds within inside the XanB catalytic site, interactions with the Asn192, Gly155, Glu173, Asp249 and Asp270 amino acids were taken into account and the interactions with glutamic and aspartic acids were considered as the most important to be analyzed.

**REFERENCES:**

1. Sanger, F., Nicklen, S. & Coulson, A. R. DNA sequencing with chain-terminating inhibitors. *Proc. Natl. Acad. Sci.* **74**, 5463–5467 (1977).

2. Goto, L. S. *et al.* Structural and functional characterization of the phosphoglucomutase from Xanthomonas citri subsp. citri. *Biochim. Biophys. Acta - Proteins Proteomics* **1864**, 1658–1666 (2016).

3. Alexandrino, A. V., Goto, L. S. & Novo-Mansur, M. T. M. TreA codifies for a trehalase with involvement in xanthomonas citri subsp. citri Pathogenicity. *PLoS One* **11**, (2016).

4. Malamud, F. *et al.* The Xanthomonas axonopodis pv. citri flagellum is required for mature biofilm and canker development. *Microbiology* **157**, 819–829 (2011).

5. O’Toole, G. A. & Kolter, R. Initiation of biofilm formation in Pseudomonas fluorescens WCS365 proceeds via multiple, convergent signalling pathways: a genetic analysis. *Mol. Microbiol.* **28**, 449–461 (1998).

6. Li, J. & Wang, N. The wxacO gene of Xanthomonas citri ssp. citri encodes a protein with a role in lipopolysaccharide biosynthesis, biofilm formation, stress tolerance and virulence. *Mol. Plant Pathol.* **12**, 381–396 (2011).

7. Laemmli, U. K. Cleavage of structural proteins during the assembly of the head of bacteriophage T4. *Nature* **227**, 680 (1970).

8. Chawla, R. *Practical clinical biochemistry: methods and interpretations*. (JP Medical Ltd, 2014).

9. Roe, J. H. A colorimetric method for the determination of fructose in blood and urine. *J. Biol. Chem.* **107**, 15–22 (1934).

10. Gracy, R. W. & Noltmann, E. A. Studies on phosphomannose isomerase I. Isolation, homogeneity measurements, and determination of some physical properties. *J. Biol. Chem.* **243**, 3161–3168 (1968).

11. Wu, B., Zhang, Y., Zheng, R., Guo, C. & Wang, P. G. Bifunctional phosphomannose isomerase/GDP- <scp>D</scp> -mannose pyrophosphorylase is the point of control for GDP- <scp>D</scp> -mannose biosynthesis in *Helicobacter pylori*. *FEBS Lett.* **519**, 87–92 (2002).

12. Davis, A. J. *et al.* Properties of GDP-mannose Pyrophosphorylase, a Critical Enzyme and Drug Target in Leishmania mexicana. *J. Biol. Chem.* **279**, 12462–12468 (2004).
